# Supplementary material for: Clostridioides difficile specific DNA adenine methyltransferase CamA squeezes and flips adenine out of DNA helix
Source: Nat Commun. 2021 Jun 8;12:3436. doi: 10.1038/s41467-021-23693-w (PMC8187626; doi:10.1038/s41467-021-23693-w)
Supplement: Supplementary file 1 — Supplementary Information [file 41467_2021_23693_MOESM1_ESM.pdf]

Supplementary Information

Zhou et al.

***Clostridioides difficile* specific DNA adenine methyltransferase CamA squeezes and flips adenine out of DNA helix**

Jujun Zhou[1,3], John R. Horton[1,3], Robert M. Blumenthal[2], Xing Zhang[1,*], Xiaodong Cheng[1,*]

[1]Department of Epigenetics and Molecular Carcinogenesis, University of Texas MD Anderson Cancer Center, Houston, TX 77030, USA

[2]Department of Medical Microbiology and Immunology, and Program in Bioinformatics, The University of Toledo College of Medicine and Life Sciences, Toledo, OH 43614, USA

[3]These authors contributed equally: J.Z. and J.R.H.

* Correspondence: XZhang21@mdanderson.org or XCheng5@mdanderson.org

Email addresses:

JZ (JZhou12@mdanderson.org); JRH (JRHorton@mdanderson.org);

RMB (Robert.Blumenthal@utoledo.edu); XZ (XZhang21@mdanderson.org);

XC (XCheng5@mdanderson.org)

Seven figures and one table

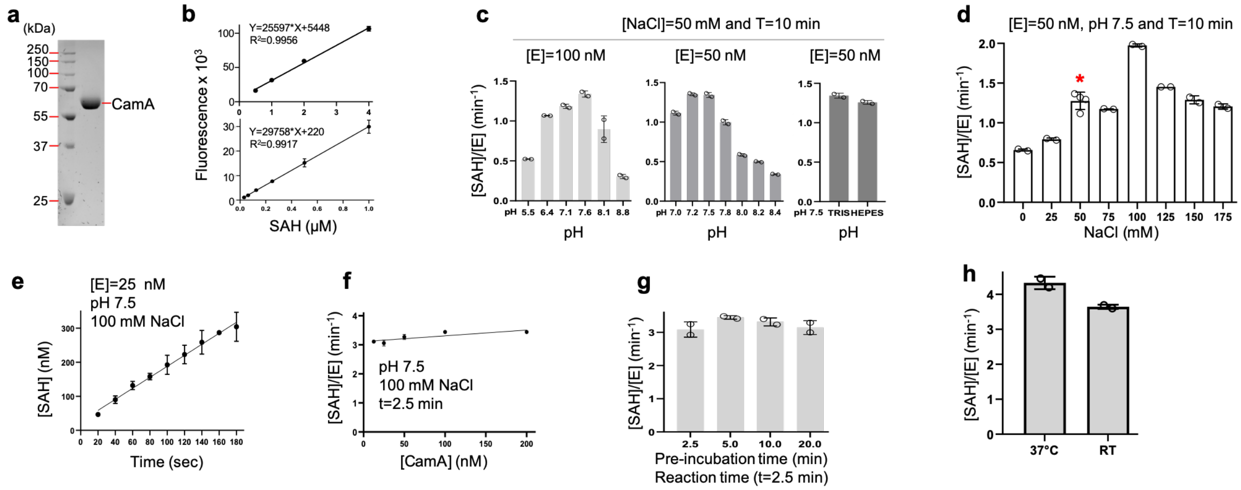

Supplementary Fig. 1 CamA enzymatic activity. **a** Example of purified recombinant CamA used in the study (12% SDS PAGE with Coomassie blue staining). We purified $N = 7$ times used for enzyme assays and co-crystallizations. **b** Calibration curves for Promega bioluminescence assay as a function of SAH concentrations in the range of above or below 1 μM (top and bottom panels) ($N = 2$). **c** pH variation ($N = 2$). **d** NaCl variation ($N = 2$ or 4 as indicated by *). **e** Reaction time variation ($N = 2$). **f** Enzyme concentration variation ($N = 2$). **g** Variation of pre-incubation time at room temperature ($N = 2$). **h** Comparison of CamA activity on DNA oligo at 37 °C and room temperature (RT; ~22 °C) ($N = 2$). For convenience, we used room temperature in this study even though CamA has a slightly higher activity at 37 °C. Source data are provided as a Source Data file.

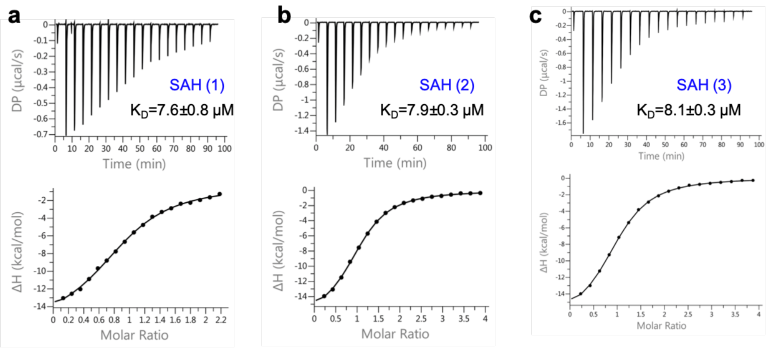

CamA	K_D (μM)	N	ΔH	-TΔS	ΔG
			ΔH-TΔS =ΔG (kcal/mol)		
SAH (1)	7.6±0.8	0.91	-15.9±0.5	8.9	-7.0
SAH (2)	7.9±0.5	1.01	-17.0±0.2	10.1	-7.0
SAH (3)	8.1±0.3	0.98	-17.4±0.2	10.5	-7.0
Sinefungin (1)	19±4	1.10	-11±1	4.9	-6.5
Sinefungin (2)	18±3	0.94	-17±2	10.4	-6.5
SAM (1)	23±10	0.88	-18±5	11.9	-6.3
SAM (2)	35±4	0.88	-24±2	18.4	-6.1

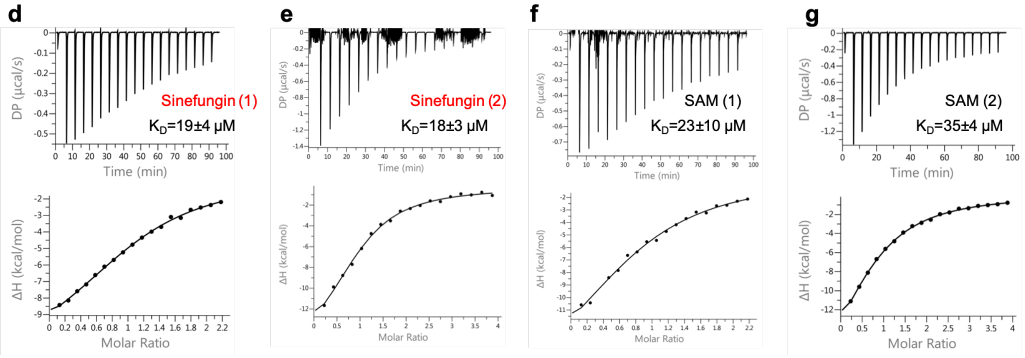

Supplementary Fig 2. Isothermal Titration Calorimetry measurements of binding affinity between CamA and cofactor. **a-c** SAH (*N* = 3) with [E]=40 μM and [SAH]=450 μM (**a**) or [E]=45 μM and [SAH]=900 μM (**b-c**). (**D-E**) Sinefungin (*N* = 2) with [E]=40 μM and [sinefungin]=450 μM (**d**) or [E]=45 μM and [sinefungin]=900 μM (**e**). **f-g** SAM (*N* = 2) with [E]=40 μM and [SAM]=450 μM (**f**) or [E]=45 μM and [SAM]=900 μM (**g**). For each experiment, top panel shows the raw microcalorimetry data. The microcalorimeter measures all heat released during the binding until the binding reaction has reached equilibrium. Note that in some experiments we have air bubbles in the sample chamber. Bottom panel shows the enthalpy (ΔH) as the amount of heat released per mole of cofactor bound (kcal/mol). Tabular summary of derived binding affinity (K_D), stoichiometry (N) and entropy and enthalpy of the binding reaction is provided for each fitting.

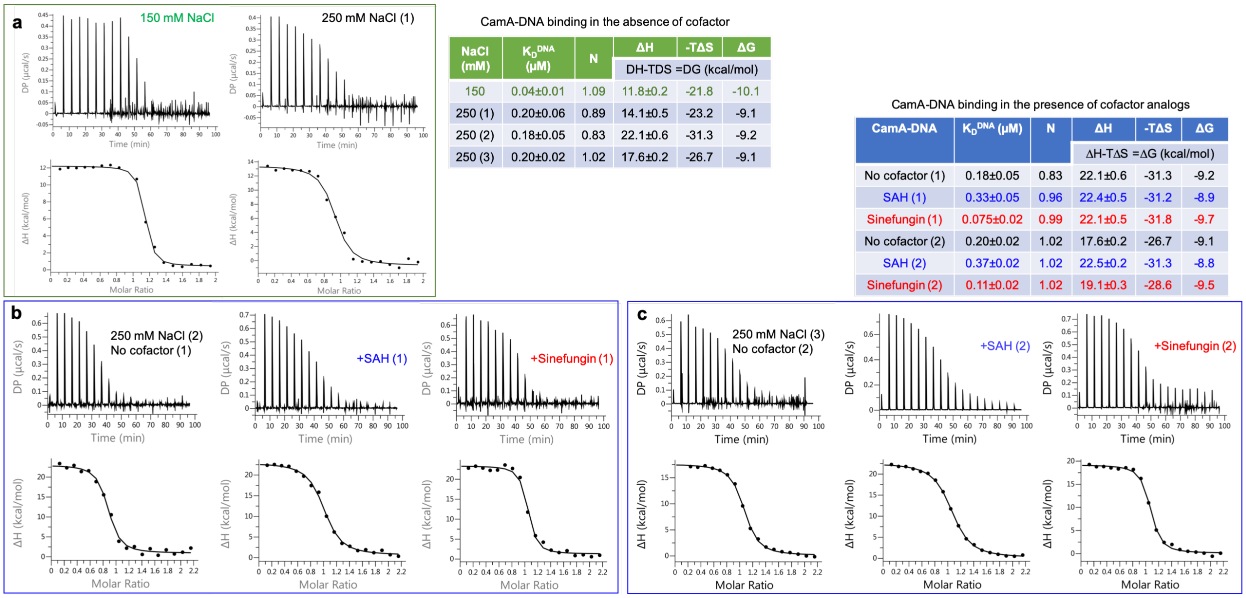

CamA-DNA binding in the absence of cofactor

NaCl (mM)	K_D^{DNA} (µM)	N	ΔH	-TΔS	ΔG
			DH-TDS =DG (kcal/mol)		
150	0.04±0.01	1.09	11.8±0.2	-21.8	-10.1
250 (1)	0.20±0.06	0.89	14.1±0.5	-23.2	-9.1
250 (2)	0.18±0.05	0.83	22.1±0.6	-31.3	-9.2
250 (3)	0.20±0.02	1.02	17.6±0.2	-26.7	-9.1

CamA-DNA binding in the presence of cofactor analogs

CamA-DNA	K_D^{DNA} (µM)	N	ΔH	-TΔS	ΔG
			ΔH-TΔS =ΔG (kcal/mol)		
No cofactor (1)	0.18±0.05	0.83	22.1±0.6	-31.3	-9.2
SAH (1)	0.33±0.05	0.96	22.4±0.5	-31.2	-8.9
Sinefungin (1)	0.075±0.02	0.99	22.1±0.5	-31.8	-9.7
No cofactor (2)	0.20±0.02	1.02	17.6±0.2	-26.7	-9.1
SAH (2)	0.37±0.02	1.02	22.5±0.2	-31.3	-8.8
Sinefungin (2)	0.11±0.02	1.02	19.1±0.3	-28.6	-9.5

Supplementary Fig 3. Isothermal Titration Calorimetry measurements of binding affinity between CamA and DNA. **a** As a function of ionic strength with [E]=20 µM and [DNA]=200 µM (*N* = 3 at 250 mM NaCl). **b-c** In the absence or presence of cofactor analog performed in 250 mM NaCl with [E]=18 µM and [DNA]=200 µM (*N* = 2). Tabular summaries of derived binding affinity (K_D), stoichiometry (N) and entropy and enthalpy of the binding reaction are provided for each fitting.

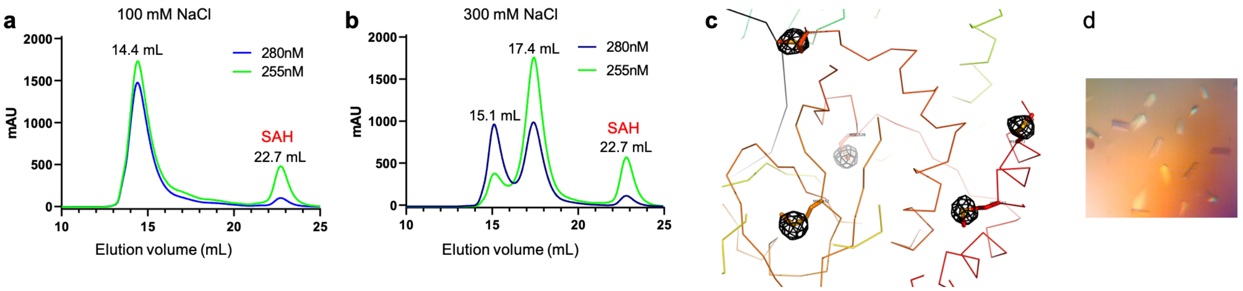

Supplementary Fig. 4. **a-b** Elution profiles of CamA-DNA complexes from sizing exclusion chromatography (Superdex 200 Increase 10/300 GL) under the buffer conditions of 100 mM NaCl (**a**) or 300 mM NaCl (**b**). The protein-DNA complex was stable under 100 mM NaCl, but SAH was dissociated. **c** The anomalous electron density, contoured at 5σ above mean, shown for five SeMet residues of the C-terminal TRD domain. **d** Graph of the SeMet-substituted CamA-DNA crystals.

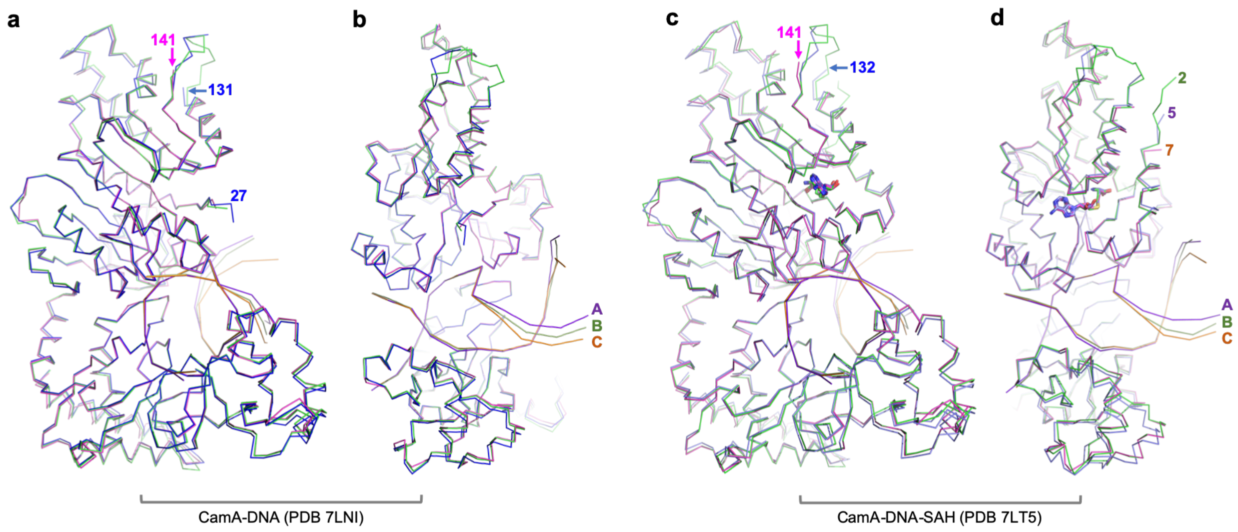

Supplementary Fig. 5. Structural comparison of three protein-DNA complexes per asymmetric unit in the space group $P2_12_12_1$. **a-b** Two views of protein-DNA complexes in the absence of SAH binding (PDB 7LNI). The largest difference in the protein components lie in the loop region 131-141 away from the protein-DNA interface (**a**), in addition to the disordered N-terminal residues (indicated by residue 27). The largest difference in the bound DNA components is located in the free end of dsDNA lacking any protein contact (**b**). **c-d** Two views of protein-DNA complexes in the presence of SAH (in stick model) (PDB 7LT5).

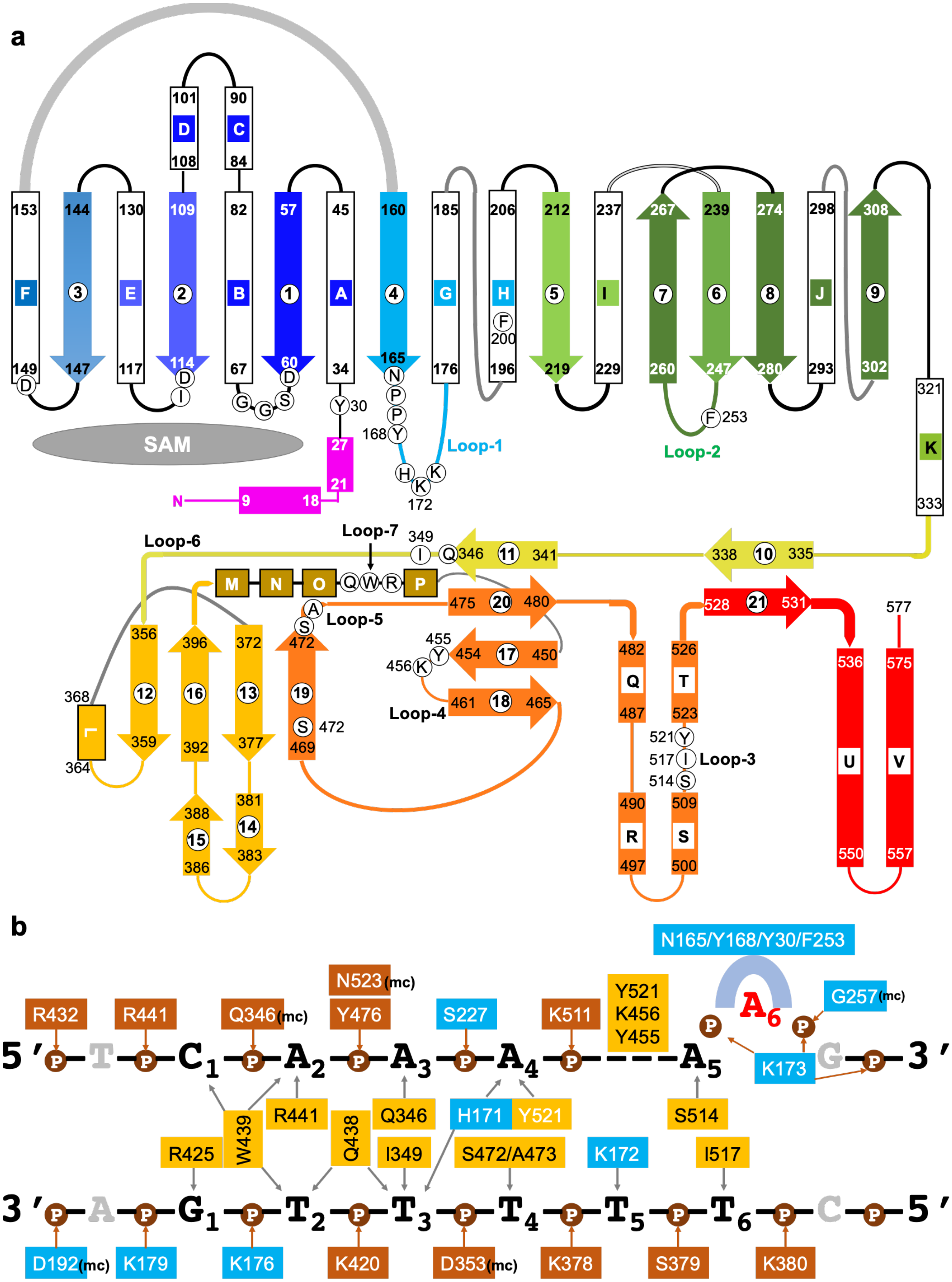

a
SAM
Loop-1
Loop-2
Loop-3
Loop-4
Loop-5
Loop-6
Loop-7
N
b
N165/Y168/Y30/F253
N523 (mc)
Y476
R432
R441
Q346 (mc)
S227
K511
Y521
K456
Y455
G257 (mc)
K173
5′ T C1 A2 A3 A4 A5 A6 G 3′
W439
R441
Q438
Q346
H171
Y521
S514
R425
I349
S472/A473
K172
I517
3′ A G1 T2 T3 T4 T5 T6 C 5′
D192 (mc)
K179
K176
K420
D353 (mc)
K378
S379
K380

Supplementary Fig. 6. **a** Schematic diagram of CamA secondary structures, with N-terminal catalytic domain (blue-to-green) and C-terminal TRD (orange-to-red). Helices (rectangles) are labeled from A to V, and strands (arrows) are labeled as 1 to 21. Functional residues, most of them are located in loops L1-to-L7, are labeled with single-letter amino acid abbreviations within white circles. The N-terminal residues undergo conformational changes upon SAH binding are shown in magenta. **b** An enlarged version of schematic of CamA-DNA interactions: residues in cyan background are from the N-terminal catalytic domain, and residues in (light and dark) orange from the C-terminal TRD. The base-specific contacts are placed between the two strands and the phosphate contacts are depicted above or below the strand. mc, main-chain-atom-mediated contacts.

Supplementary Table 1. Summary of X-ray data collection and refinement statistics (*)

CamA	SeMet+DNA	WT+DNA	WT+DNA+SAH
PDB ID	7LNI	7LNJ	7LT5
Wavelength (Å)	0.97795	1.00000	1.00000
Space group	$P2_12_12_1$	$P2_12_12_1$	$P2_12_12_1$
Cell dimensions (Å)	82.07, 160.99, 231.32	82.25, 161.36, 231.63	81.42, 160.86, 230.16
Resolution (Å)	40.76-2.69 (2.79-2.68)	44.35-2.69 (2.79-2.68)	45.10-2.54 (2.64-2.54)
[a] R_{merge}	0.308 (0.902)	0.224 (0.940)	0.179 (0.948)
R_{pim}	0.053 (0.515)	0.068 (0.690)	0.058 (0.478)
$CC_{1/2}$	(0.671)	(0.384)	(0.564)
[b] <I/σI>	16.1 (1.7)	16.3 (0.9)	10.7 (1.4)
Completeness (%)	99.9 (99.6)	87.8 (73.8)	88.8 (83.1)
Redundancy	32.9 (23.8)	10.3 (7.0)	9.1 (8.7)
Observed reflections	2,825,594	783,940	802,514
Unique reflections	85,923 (8,447) (78,411 have both I+ and I-)	76,108 (6,312)	88,557 (8,168)
Mean FOM (SAD)	0.238		
R-Factor (density modification)	0.24		
**Refinement**			
Resolution (Å)	2.68	2.68	2.54
No. reflections	164,196 (85,819 non-anomalous)	75,973	88,478
[c] R_{work} / [d] R_{free}	0.203 / 0.223	0.210 / 0.251	0.176/0.212
No. Atoms			
Protein	13,371	13,247	13,908
DNA	1704	1686	1686
Solvent	278	124	371
SAH	-	-	78
B Factors (Å^2)			
Protein	55.7	63.4	49.5
DNA	60.3	67.3	55.1
Solvent	39.0	45.3	37.8
SAH	-	-	47.8
**R.m.s. deviations**			
Bond lengths (Å)	0.002	0.002	0.002
Bond angles (˚)	0.4	0.4	0.5

* Values in parenthesis correspond to highest resolution shell;

[a] $R_{merge}=\Sigma|I-\langle I\rangle|/\Sigma I$, where I is the observed intensity and <I> is the averaged intensity from multiple observations;

[b] <I/σI> =averaged ratio of the intensity (I) to the error of the intensity (σI);

[c] $R_{work}=\Sigma|Fo-Fc|/\Sigma|Fo|$, where Fo and Fc are the observed and calculated structure factors;

[d] R_{free} was calculated using a randomly chosen subset (5%) of the reflections not used in refinement.

Supplementary Fig. 7. Sequence alignment of CamA orthologs (see following pages).

Only compared CamA orthologs that were nonidentical to the query, in order to more sensitively detect sequence variation. Substitutions are highlighted in cyan, DNA recognition residues in magenta, and universally-conserved residues (with roles as labeled and described in the text) in yellow. The boundary between the catalytic domain and target recognition domain (TRD) is also indicated.

Supplementary Fig. 7. Sequence alignment of CamA orthologs. (Query=reference 630 genome)

```
                     1        10        20        30        40        50       60 motif I70         80        90        100
                     |        |         |    p    as        |         |         | sssss   |         |         |         |
Query           1    MDDISQDNFLLSKEYENSLDVDTKKASGIYYTPKIIVDYIVKKTLKNHDIIKNPYPRILDISCGCGNFLLEVYDILYDLFEENIYELKKKYDENYWTVDN 100
WP_054274745.1  1    MDDISQDNFLLSKEYENSLDVDTKKASGIYYTPKIIVDYIVKKTLKNHDIIKNPYPRILDISCGCGNFLLEVYDILYDLFEENIYELKKKYDENYWTVDN 100
VIB38147.1      1    MDDISQDNFLLSKEYENSLDVDTKKASGIYYTPKIIVDYIVKKTLKNHDIIKNPYPRILDISCGCGNFLLEVYDILYDLFEENIYELKKKYDENYWTVDN 100
WP_009906092.1  1    MDDISQDNFLLSKEYENSLDVDTKKASGIYYTPKIIVDYIVKKTLKNHDIIKNPYPRILDISCGCGNFLLEVYDILYDLFEENIYELKKKYDENYWTVDN 100
WP_003422891.1  1    MDDISQDNFLLSKEYENSLDVDTKKASGIYYTPKIIVDYIVKKTLKNHDIIKNPYPRILDISCGCGNFLLEVYDILYDLFEENIYELKKKYDENYWTVDN 100
WP_107616100.1  1    MDDISQDNFLLSKEYENSLDVDTKKASGIYYTPKIIVDYIVKKTLKNHDIIKNPYPRILDISCGCGNFLLEVYDILYDLFEENIYELKKKYDENYWTVDN 100
VIF75342.1      1    MDDISQDNFLLSKEYENSLDVDTKKASGIYYTPKIIVDYIVKKTLKNHDIIKNPYPRILDISCGCGNFLLEVYDILYDLFEENIYELKKKYDESYWTVDN 100
WP_107619286.1  1    MDDISQDNFLLSKEYENSLDVDTKKASGIYYTPKIIVDYIVKKTLKNHDIIKNPYPRILDISCGCGNFLLEVYDILYDLFEENIYKLKKKYDENYWTVDN 100
WP_133143045.1  1    MDDISQDNFLLSKEYENSLDVDTKKASGIYYTPKIIVDYIVKKTLKNHDIIKNPYPRILDISCGCGNFLLEVYDILYDLFEENIYELKKKYDESYWTVDN 100
EGT2204202.1    1    MDDISQDNFLLSKEYENSLDVDTKKASGIYYTPKIIVDYIVKKTLKNHDIIKNPYPRILDISCGCGNFLLEVYDILYDLFEENIYELKKKYDENYWTVDN 100
WP_131040770.1  1    MDDISQDNFLLSKEYENSLDVDTKKASGIYYTPKIIVDYIVKKTLKNHDIIKNPYPRILDISCGCGNFLLEVYDILYDLFEENIYELKKKYDENYWTVDN 100
WP_021385685.1  1    MDDISQDNFLLSKEYENSLDVDTKKASGIYYTPKIIVDYIVKKTLKNHDIIKNPYPRILDISCGCGNFLLEVYDILYDLFEENIYELKEKYDENYWTVNN 100
WP_054277349.1  1    MDDISQDNFLLSKEYENSLDVDTKKASGIYYTPKIIVDYIVKKTLKNHDIIKNPYPRILDISCGCGNFLLEVYDILYDLFEENIYELKEKYDETYWTVDN 100
WP_167640826.1  1    MDDISQDNFLLSKEYENSLDVDTKKASGIYYTPKIIVDYIVKKTLKNHDIIKNPYPRILDISCGCGNFLLEVYDILYDLFEENIYELKEKYDEAYWTVDN 100
WP_074178811.1  1    MDDISQDNFLLSKEYENSLDVDTKKASGIYYTPKIIVDYIVKKTLKNHDIIKNPYPRILDISCGCGNFLLEVYDILYDLFEENIYELKEKYDEAYWTVDN 100
NJI79135.1      1    MDDISQDNFLLSKEYENSLDVDTKKASGIYYTPKIIVDYIVKKTLKNHDIIKNPYPRILDISCGCGNFLLEVYDILYDLFEENIYELKEKYDEAYWTVDN 100
WP_169469916.1  1    MDDISQDNFLLSKEYENSLDVDTKKAGGIYYTPKIIVDYIVKKTLKSHDIIKNPYPRILDISCGCGNFLLEVYDILYDLFEENIYELKEKYDETYWTVDN 100
WP_054271332.1  1    MDDISQDNFLLSKEYENSLDVDTKKASGIYYTPKIIVDYIVKKTLKNHDIIKNPYPRILDISCGCGNFLLEVYDILYDLFEENIYELKEKYDEAYWTVDN 100
EGT4516689.1    1    MDDISQDNFLLSKEYENSLDVDTKKASGIYYTPKIIVDYIVKKTLKNHDIIKNPYPRILDISCGCGNFLLEVYDILYDLFEENIYELKKKYDENYWTVDN 100
MBH7490777.1    1    MDDISQDNFLLSKEYENSLDVDTKKASGIYYTPKIIVDYIVKKTLKNHDIIKNPYPRILDISCGCGNFLLEVYDILYDLFEENIYELKKKYDENYWTVDN 100
EGT4155821.1    1    MDDISQDNFLLSKEYENSLDVDTKKASGIYYTPKIIVDYIVKKTLKNHDIIKNPYPRILDISCGCGNFLLEVYDILYDLFEENIYELKKKYDENYWTVDN 100
MBH7182547.1    1    MDDISQDNFLLSKEYENSLDVDTKKASGIYYTPKIIVDYIVKKTLKNHDIIKNPYPRILDISCGCGNFLLEVYDILYDLFEENIYELKKKYDENYWTVDN 100
MBF9877285.1    1    MDDISQDNFLLSKEYENSLDVDTKKASGIYYTPKIIVDYIVKKTLKNHDIIKNPYPRILDISCGCGNFLLEVYDILYDLFEENIYELKKKYDENYWTVDN 100
MBG0179561.1    1    MDDISQDNFLLSKEYENSLDVDTKKASGIYYTPKIIVDYIVKKTLKNHDIIKNPYPRILDISCGCGNFLLEVYDILYDLFEENIYELKKKYDENYWTVDN 100
MBG0197089.1    1    MDDISQDNFLLSKEYENSLDVDTKKASGIYYTPKIIVDYIVKKTLKNHDIIKNPYPRILDISCGCGNFLLEVYDILYDLFEENIYELKKKYDENYWTVDN 100
EGT3970705.1    1    MDDISQDNFLLSKEYENSLDVDTKKASGIYYTPKIIVDYIVKKTLKNHDIIKNPYPRILDISCGCGNFLLEVYDILYDLFEENIYELKKKYDENYWTVDN 100
MBG0131288.1    1    MDDISQDNFLLSKEYENSLDVDTKKASGIYYTPKIIVDYIVKKTLKNHDIIKNPYPRILDISCGCGNFLLEVYDILYDLFEENIYELKKKYDENYWTVDN 100
EGT4149356.1    1    MDDISQDNFLLSKEYENSLDVDTKKASGIYYTPKIIVDYIVKKTLKNHDIIKNPYPRILDISCGCGNFLLEVYDILYDLFEENIYELKKKYDENYWTVDN 100
EGT3786504.1    1    MDDISQDNFLLSKEYENSLDVDTKKASGIYYTPKIIVDYIVKKTLKNHDIIKNPYPRILDISCGCGNFLLEVYDILYDLFEENIYELKKKYDENYWTVDN 100
HAU4954234.1    1    MDDISQDNFLLSKEYENSLDVDTKKASGIYYTPKIIVDYIVKKTLKNHDIIKNPYPRILDISCGCGNFLLEVYDILYDLFEENIYELKKKYDENYWIVDN 100
WP_131072671.1  1    MDDISQDNFLLSKEYENSLDVDTKKASGIYYTPKIIVDYIVKKTLKNHDIIKNPYPRILDISCGCGNFLLEVYDILYDLFEENIYELKKKYDENYWTVDN 100
EGT4119714.1    1    MDDISQDNFLLSKEYENSLDVDTKKASGIYYTPKIIVDYIVKKTLKNHDIIKNPYPRILDISCGCGNFLLEVYDILYDLFEENIYELKKKYDENYWTVDN 100
WP_131036696.1  1    MDDISQDNFLLSKEYENSLDVDTKKASGIYYTPKIIVDYIVKKTLKNHDIIKNPYPRILDISCGCGNFLLEVYDILYDLFEENIYELKKKYDENYWTVDN 100
EGT5091780.1    1    MDDISQDNFLLSKEYENSLDVDTKKASGIYYTPKIIVDYIVKKTLKNHDIIKNPYPRILDISCGCGNFLLEVYDILYDLFEENIYELKKKYDENYWTVDN 100
HAT4772283.1    1    MDDISQDNFLLSKEYENSLDVDTKKASGIYYTPKIIVDYIVKKTLKNHDIIKNPYPRILDISCGCGNFLLEVYDILYDLFEENIYELKKKYDENYWTVDN 100
EGT3843784.1    1    MDDISQDNFLLSKEYENSLDVDTKKASGIYYTPKIIVDYIVKKTLKNHDIIKNPYPRILDISCGCGNFLLEVYDILYDLFEENIYELKKKYDENYWTVDN 100
WP_095903253.1  1    MDDISQDNFLLSKEYENSLDVDTKKASGIYYTPKIIVDYIVKKTLKNHDIIKNPYPRILDISCGCGNFLLEVYDILYDLFEENIYELKKKYDENYWTVDN 100
WP_131064843.1  1    MDDISQDNFLLSKEYENSLDVDTKKASGIYYTPKIIVDYIVKKTLKNHDIIKNPYPRILDISCGCGNFLLEVYDILYDLFEENIYELKKKYDENYWTVDN 100
EGT3983209.1    1    MDDISQDNFLLSKEYENSLDVDTKKASGIYYTPKIIVDYIVKKTLKNHDIIKNPYPRILDISCGCGNFLLEVYDILYDLFEENIYELKKKYDENYWTVDN 100
HAU5286908.1    1    MDDISQDNFLLSKEYENSLDVDTKKASGIYYTPKIIVDYIVKKTLKNHDIIKNPYPRILDISCGCGNFLLEVYDILYDLFEENIYELKKKYDENYWTVDN 100
EGT5172991.1    1    MDDISQDNFLLSKEYENSLDVDTKKASGIYYTPKIIVDYIVKKTLKNHDIIKNPYPRILDISCGCGNFLLEVYDILYDLFEENIYELKKKYDENYWTVDN 100
WP_021405820.1  1    MDDISQDNFLLSKEYENSLDVDTKKASGIYYTPKIIVDYIVKKTLKNHDIIKNPYPRILDISCGCGNFLLEVYDILYDLFEENIYELKKKYDENYWTVDN 100
EGT3776579.1    1    MDDISQDNFLLSKEYENSLDVDTKKASGIYYTPKIIVDYIVKKTLKNHDIIKNPYPRILDISCGCGNFLLEVYDILYDLFEENIYELKKKYDENYWTVDN 100
HAT4798010.1    1    MDDISQDNFLLSKEYENSLDVDTKKASGIYYTPKIIVDYIVKKTLKNHDIIKNPYPRILDISCGCGNFLLEVYDILYDLFEENIYELKKKYDENYWTVDN 100
HAT4801762.1    1    MDDISQDNFLLSKEYENSLDVDTKKASGIYYTPKIIVDYIVKKTLKNHDIIKNPYPRILDISCGCGNFLLEVYDILYDLFEENIYELKKKYDENYWTVDN 100
EGT4089306.1    1    MDDISQDNFLLSKEYENSLDVDTKKASGIYYTPKIIVDYIVKKTLKNHDIIKNPYPRILDISCGCGNFLLEVYDILYDLFEENIYELKKKYDENYWTVDN 100
EGT3949436.1    1    MDDISQDNFLLSKEYENSLDVDTKKASGIYYTPKIIVDYIVKKTLKNHDIIKNPYPRILDISCGCGNFLLEVYDILYDLFEENIYELKKKYDENYWIVDN 100
EGT4921482.1    1    MDDISQDNFLLSKEYENSLDVDTKKASGIYYTPKIIVDYIVKKTLKNHDIIKNPYPRILDISCGCGNFLLEVYDILYDLFEENIYELKKKYDENYWTVDN 100
EGT4197963.1    1    MDDISQDNFLLSKEYENSLDVDTKKASGIYYTPKIIVDYIVKKTLKNHDIIKNPYPRILDISCGCGNFLLEVYDILYDLFEENIYELKKKYDENYWTVDN 100
EGT4973643.1    1    MDDISQDNFLLSKEYENSLDVDTKKASGIYYTPKIIVDYIVKKTLKNHDIIKNPYPRILDISCGCGNFLLEVYDILYDLFEENIYELKKKYDENYWTVDN 100
WP_095905217.1  1    MDDISQDNFLLSKEYENSLDVDTKKASGIYYTPKIIVDYIVKKTLKNHDIIKNPYPRILDISCGCGNFLLEVYDILYDLFEENIYELKKKYDENYWTVDN 100
WP_021380625.1  1    MDDISQDNFLLSKEYENSLDVDTKKASGIYYTPKIIVDYIVKKTLKNHDIIKNPYPRILDISCGCGNFLLEVYDILYDLFEENIYELKKKYDENYWTVDN 100
HAU4836842.1    1    MDDISQDNFLLSKEYENSLDVDTKKASGIYYTPKIIVDYIVKKTLKNHDIIKNPYPRILDISCGCGNFLLEVYDILYDLFEENIYELKKKYDENYWTVDN 100
WP_167652924.1  1    MDDISQDNFLLSKEYENSLDVDTKKASGIYYTPKIIVDYIVKKTLKNHDIIKNPYPRILDISCGCGNFLLEVYDILYDLFEENIYELKKKYDENYWTVDN 100
WP_167626365.1  1    MDDISQDNFLLSKEYENSLDVDTKKASGIYYTPKIIVDYIVKKTLKNHDIIKNPYPRILDISCGCGNFLLEVYDILYDLFEENIYELKKKYDENYWTVDN 100
EGT5068818.1    1    MDDISQDNFLLSKEYENSLDVDTKKASGIYYTPKIIVDYIVKKTLKNHDIIKNPYPRILDISCGCGNFLLEVYDILYDLFEENIYELKKKYDENYWTVDN 100
WP_131043637.1  1    MDDISQDNFLLSKEYENSLDVDTKKASGIYYTPKIIVDYIVKKTLKNHDIIKNPYPRILDISCGCGNFLLEVYDILYDLFEENIYELKKKYDENYWIVDN 100
WP_095891409.1  1    MDDISQDNFLLSKEYENSLDVDTKKASGIYYTPKIIVDYIVKKTLKNHDIIKNPYPRILDISCGCGNFLLEVYDILYDLFEENIYELKKKYDENYWTVDN 100
```

```
                    101       110       120       130       140       150       160   IV  170       180       190       200
                      |         |   ss    |         |         |         s|        |     aaaa |bpp  p sp|         |         s
Query           101 IHRHILNYCIYGADIDEKAISILKDSLTNKKVVNDLDESDIKINLFCCDSLKKKWRYKFDYIVGNPPYIGHKKLEKKYKKFLLEKYSEVYKDKADLYFCF 200
WP_054274745.1  101 IHRHILNYCIYGADIDEKAIKILKDSLRNKKVVNDLDESDIKINLFCCDSLKKNWRYKFDYIIGNPPYIGHKKLEKKYKKFLVEKYSEVYKDKADLYFCF 200
VIB38147.1      101 IHSHILNYCIYGADIDEKAISILKDSLINKKVVNDLDESDIKINLFCCDSLKKKWRYKFDYIVGNPPYIGHKKLEKKYKKFLLEKYSEVYKDKADLYFCF 200
WP_009906092.1  101 IHSHILNYCIYGADIDEKAISILKDSLINKKVVNDLDESDIKINLFCCDSLKKKWRYKFDYIVGNPPYIGHKKLEKKYKKFLLEKYSEVYKDKADLYFCF 200
WP_003422891.1  101 IHSHILNYCIYGADIDEKAISILKDSLINKKVVNDLDESDIKINLFCCDSLKKKWRYKFDYIVGNPPYIGHKKLEKKYKKFLLEKYSEVYKDKADLYFCF 200
WP_107616100.1  101 IHSHILNYCIYGADIDEKAISILKDSLINKKVVNDLDESDIKINLFCCDSLKKKWRYKFDYIVGNPPYIGHKKLEKKYKKFLLEKYSEVYKDKADLYFCF 200
VIF75342.1      101 IHSHILNYCIYGADIDEKAISILKDSLINKKVVNDLDESDIKINLFCCDSLKKKWRYKFDYIVGNPPYIGHKKLEKKYKKFLLEKYSEVYKDKADLYFCF 200
WP_107619286.1  101 IHSHILNYCIYGADIDEKAISILKDSLINKKVVNDLDESDIKINLFCCDSLKKKWRYKFDYIVGNPPYIGHKKLEKKYKKFLLEKYSEVYKDKADLYFCF 200
WP_133143045.1  101 IHSHILNYCIYGADIDEKAISILKDSLINKKVVNDLDESDIKINLFCCDSLKKKWRYKFDYIVGNPPYIGHKKLEKKYKKFLLEKYSEVYKDKADLYFCF 200
EGT2204202.1    101 IHSHILNYCIYGADIDEKAISILKDSLINKKVVNDLDESDIKINLFCCDSLKKKWRYKFDYIVGNPPYIGHKKLEKKYKKFLLEKYSEVYKDKADLYFCF 200
WP_131040770.1  101 IHSHILNYCIYGADIDEKAISILKDSLINKKVVNDLDESDIKINLFCCDSLKKKWRYKFDYIVGNPPYIGHKKLEKKYKKFLLEKYSEVYKDKADLYFCF 200
WP_021385685.1  101 IHSHILNYCIYGADIDEKAINILKDSLINKKAVNDLEESDIKINLFCCDSLKKNWRYKFDYIIGNPPYIGHKKLEKKYKKFLLEKYSEVYKDKADLYFCF 200
WP_054277349.1  101 IHSHILNYCIYGADIDEKAINILKDSLINKKAVNDLEESDIKINLFCCDSLKKNWRYKFDYIIGNPPYIGHKKLEKKYKKFLLEKYNEVYKDKADLYFCF 200
WP_167640826.1  101 IHSHILNYCIYGADIDEKAINILKDSLINKKAINDLEESNIKINLFCCDSLKKNWRYKFDYIIGNPPYIGHKKLEKKYKKFLLEKYSEVYKDKADLYFCF 200
WP_074178811.1  101 IHNHILNYCIYGADIDEKAINILKDSLINKKAINDLEESNIKINLFCCDSLKKNWRYKFDYIIGNPPYIGHKKLEKKYKKFLLEKYSEVYKDKADLYFCF 200
NJI79135.1      101 IHSHILNYCIYGADIDEKAINILKDSLINKKAINDLEESNIKINLFCCDSLKKNWRYKFDYIIGNPPYIGHKKLEKKYKKFLLEKYSEVYKDKADLYFCF 200
WP_169469916.1  101 IHSHILNYCIYGADIDEKAINILKDSLINKKAINDLGESDIKINLFCCDSLKKNWRYKFDYIIGNPPYIGHKKLEKKYKKFLIEKYSEVYKDKADLYFCF 200
WP_054271332.1  101 IHSHILNYCIYGADIDEKAINILKDSLINKKAINDLEESNIKINLFCCDSLKKNWRYKFDYIIGNPPYIGHKKLEKKYKKFLLEKYSEVYKDKADLYFCF 200
EGT4516689.1    101 IHRHILNYCIYGADIDEKAISILKDSLTNKKVVNDLDESDIKINLFCCDSLKKKWRYKFDYIVGNPPYIGHKKLEKKYKKFLLEKYSEVYKDKADLYFCF 200
MBH7490777.1    101 IHRHILNYCIYGADIDEKAISILKDSLTNKKVVNDLDESDIKINLFCCDSLKKKWRYKFDYIVGNPPYIGHKKLEKKYKKFLLEKYSEVYKDKADLYFCF 200
EGT4155821.1    101 IHRHILNYCIYGADIDEKAISILKDSLTNKKVVNDLDESDIKINLFCCDSLKKKWRYKFDYIVGNPPYIGHKKLEKKYKKFLLEKYSEVYKDKADLYFCF 200
MBH7182547.1    101 IHRHILNYCIYGADIDEKAISILKDSLTNKKVVNDLDESDIKINLFCCDSLKKKWRYKFDYIVGNPPYIGHKKLEKKYKKFLLEKYSEVYKDKADLYFCF 200
MBF9877285.1    101 IHRHILNYCIYGADIDEKAISILKDSLTNKKVVNDLDESDIKINLFCCDSLKKKWRYKFDYIVGNPPYIGHKKLEKKYKKFLLEKYSEVYKDKADLYFCF 200
MBG0179561.1    101 IHRHILNYCIYGADIDEKAISILKDSLTNKKVVNDLDESDIKINLFCCDSLKKKWRYKFDYIVGNPPYIGHKKLEKKYKKFLLEKYSEVYKDKADLYFCF 200
MBG0197089.1    101 IHRHILNYCIYGADIDEKAISILKDSLTNKKVVNDLDESDIKINLFCCDSLKKKWRYKFDYIVGNPPYIGHKKLEKKYKKFLLEKYSEVYKDKADLYFCF 200
EGT3970705.1    101 IHRHILNYCIYGADIDEKAISILKDSLTNKKVVNDLDESDIKINLFCCDSLKKKWRYKFDYIVGNPPYIGHKKLEKKYKKFLLEKYSEVYKDKADLYFCF 200
MBG0131288.1    101 IHRHILNYCIYGADIDEKAISILKDSLTNKKVVNDLDESDIKINLFCCDSLKKKWRYKFDYIVGNPPYIGHKKLEKKYKKFLLEKYSEVYKDKADLYFCF 200
EGT4149356.1    101 IHRHILNYCIYGADIDEKAISILKDSLTNKKVVNDLDESDIKINLFCCDSLKKKWRYKFDYIVGNPPYIGHKKLEKKYKKFLLEKYSEVYKDKADLYFCF 200
EGT3786504.1    101 IHRHILNYCIYGADIDEKAISILKDSLTNKKVVNDLDESDIKINLFCCDSLKKKWRYKFDYIVGNPPYIGHKKLEKKYKKFLLEKYSEVYKDKADLYFCF 200
HAU4954234.1    101 IHRHILNYCIYGADIDEKAISILKDSLTNKKVVNDLDESDIKINLFCCDSLKKKWRYKFDYIVGNPPYIGHKKLEKKYKKFLLEKYSEVYKDKADLYFCF 200
WP_131072671.1  101 IHRHILNYCIYGADIDEKAISILKDSLTNKKVVNDLDESDIKINLFCCDSLKKKWRYKFDYIVGNPPYIGHKKLEKKYKKFLLEKYSEVYKDKADLYFCF 200
EGT4119714.1    101 IHRHILNYCIYGADIDEKAISILKDSLTNKKVVNDLDESDIKINLFCCDSLKKKWRYKFDYIVGNPPYIGHKKLEKKYKKFLLEKYSEVYKDKADLYFCF 200
WP_131036696.1  101 IHRHILNYCIYGADIDEKAISILKDSLTNKKVVNDLDESDIKINLFCCDSLKKKWRYKFDYIVGNPPYIGHKKLEKKYKKFLLEKYSEVYKDKADLYFCF 200
EGT5091780.1    101 IHRHILNYCIYGADIDEKAISILKDSLTNKKVVNDLDESDIKINLFCCDSLKKKWRYKFDYIVGNPPYIGHKKLEKKYKKFLLEKYSEVYKDKADLYFCF 200
HAT4772283.1    101 IHRHILNYCIYGADIDEKAISILKDSLTNKKVVNDLDESDIKINLFCCDSLKKKWRYKFDYIVGNPPYIGHKKLEKKYKKFLLEKYSEVYKDKADLYFCF 200
EGT3843784.1    101 IHRHILNYCIYGADIDEKAISILKDSLTNKKVVNDLDESDIKINLFCCDSLKKKWRYKFDYIVGNPPYIGHKKLEKKYKKFLLEKYSEVYKDKADLYFCF 200
WP_095903253.1  101 IHRHILNYCIYGADIDEKAISILKDSLTNKKVVNDLDESDIKINLFCCDSLKKKWRYKFDYIVGNPPYIGHKKLEKKYKKFLLEKYSEVYKDKADLYFCF 200
WP_131064843.1  101 IHRHILNYCIYGADIDEKAISILKDSLTNKKVVNDLDESDIKINLFCCDSLKKKWRYKFDYIVGNPPYIGHKKLEKKYKKFLLEKYSEVYKDKADLYFCF 200
EGT3983209.1    101 IHRHILNYCIYGADIDEKAISILKDSLTNKKVVNDLDESDIKINLFCCDSLKKKWRYKFDYIVGNPPYIGHKKLEKKYKKFLLEKYSEVYKDKADLYFCF 200
HAU5286908.1    101 IHSHILNYCIYGADIDEKAISILKDSLINKKVVNDLDESDIKINLFCCDSLKKKWRYKFDYIVGNPPYIGHKKLEKKYKKFLLEKYSEVYKDKADLYFCF 200
EGT5172991.1    101 IHRHILNYCIYGADIDEKAISILKDSLTNKKVVNDLDESDIKINLFCCDSLKKKWRYKFDYIVGNPPYIGHKKLEKKYKKFLLEKYSEVYKDKADLYFCF 200
WP_021405820.1  101 IHRHILNYCIYGADIDEKAISILKDSLTNKKVVNDLDESDIKINLFCCDSLKKKWRYKFDYIVGNPPYIGHKKLEKKYKKFLLEKYSEVYKDKADLYFCF 200
EGT3776579.1    101 IHRHILNYCIYGADIDEKAISILKDSLTNKKVVNDLDESDIKINLFCCDSLKKKWRYKFDYIVGNPPYIGHKKLEKKYKKFLLEKYSEVYKDKADLYFCF 200
HAT4798010.1    101 IHRHILNYCIYGADIDEKAISILKDSLTNKKVVNDLDESDIKINLFCCDSLKKKWRYKFDYIVGNPPYIGHKKLEKKYKKFLLEKYSEVYKDKADLYFCF 200
HAT4801762.1    101 IHRHILNYCIYGADIDEKAISILKDSLTNKKVVNDLDESDIKINLFCCDSLKKKWRYKFDYIVGNPPYIGHKKLEKKYKKFLLEKYSEVYKDKADLYFCF 200
EGT4089306.1    101 IHRHILNYCIYGADIDEKAISILKDSLTNKKVVNDLDESDIKINLFCCDSLKKKWRYKFDYIVGNPPYIGHKKLEKKYKKFLLEKYSEVYKDKADLYFCF 200
EGT3949436.1    101 IHRHILNYCIYGADIDEKAISILKDSLTNKKVVNDLDESDIKINLFCCDSLKKKWRYKFDYIVGNPPYIGHKKLEKKYKKFLLEKYSEVYKDKADLYFCF 200
EGT4921482.1    101 IHRHILNYCIYGADIDEKAISILKDSLTNKKVVNDLDESDIKINLFCCDSLKKKWRYKFDYIVGNPPYIGHKKLEKKYKKFLLEKYSEVYKDKADLYFCF 200
EGT4197963.1    101 IHRHILNYCIYGADIDEKAISILKDSLTNKKVVNDLDESDIKINLFCCDSLKKKWRYKFDYIVGNPPYIGHKKLEKKYKKFLLEKYSEVYKDKADLYFCF 200
EGT4973643.1    101 IHRHILNYCIYGADIDEKAISILKDSLTNKKVVNDLDESDIKINLFCCDSLKKKWRYKFDYIVGNPPYIGHKKLEKKYKKFLLEKYSEVYKDKADLYFCF 200
WP_095905217.1  101 IHRHILNYCIYGADIDEKAISILKDSLTNKKVVNDLDESDIKINLFCCDSLKKKWRYKFDYIVGNPPYIGHKKLEKKYKKFLLEKYSEVYKDKADLYFCF 200
WP_021380625.1  101 IHRHILNYCIYGADIDEKAISILKDSLTNKKVVNDLDESDIKINLFCCDSLKKKWRYKFDYIVGNPPYIGHKKLEKKYKKFLLEKYSEVYKDKADLYFCF 200
HAU4836842.1    101 IHRHILNYCIYGADIDEKAISILKDSLTNKKVVNDLDESDIKINLFCCDSLKKKWRYKFDYIVGNPPYIGHKKLEKKYKKFLLEKYSEVYKDKADLYFCF 200
WP_167652924.1  101 IHRHILNYCIYGADIDEKAISILKDSLTNKKVVNDLDESDIKINLFCCDSLKKKWRYKFDYIVGNPPYIGHKKLEKKYKKFLLEKYSEVYKDKADLYFCF 200
WP_167626365.1  101 IHRHILNYCIYGADIDEKAISILKDSLTNKKVVNDLDESDIKINLFCCDSLKKKWRYKFDYIVGNPPYIGHKKLEKKYKKFLLEKYSEVYKDKADLYFCF 200
EGT5068818.1    101 IHRHILNYCIYGADIDEKAISILKDSLTNKKVVNDLDESDIKINLFCCDSLKKKWRYKFDYIVGNPPYIGHKKLEKKYKKFLLEKYSEVYKDKADLYFCF 200
WP_131043637.1  101 IHRHILNYCIYGADIDEKAISILKDSLTNKKVVNDLDESDIKINLFCCDSLKKKWRYKFDYIVGNPPYIGHKKLEKKYKKFLLEKYSEVYKDKADLYFCF 200
WP_095891409.1  101 IHRHILNYCIYGADIDEKAISILKDSLTNKKVVNDLDESDIKINLFCCDSLKKKWRYKFDYIVGNPPYIGHKKLEKKYKKFLLEKYSEVYKDKADLYFCF 200
```

```
                        201        210         220         230         240         250         260         270         280         290         300
                          |          |           |      p    |           |          |a cage    |           |           |           |           |
Query             201   YKKIIDILKQGGIGSVITPRYFLESLSGKDLREYIKSNVNVQEIVDFLGANIFKNIGVSSCILTFDKKKTKETYIDVFKIKNEDICINKFETLEELLKSS  300
WP_054274745.1    201   YKKIIDILKKGGTGSVITPRYFLESLSGKDLREYIKSNVNVKEIVDFLGANIFKNIGVSSCILTFDKKKSKDTPIDVFKIKNEDICINKFETLEELLNSS  300
VIB38147.1        201   YKKIIDILKQGGIGSVITPRYFLESLSGKDLREYIKSNVNVQEIVDFLGANIFKNIGVSSCILTFDKKKTKKTYIDVFKIKNEDICINKFETLEELLKSS  300
WP_009906092.1    201   YKKIIDILKQGGIGSVITPRYFLESLSGKDLREYIKSNVNVQEIVDFLGANIFKNIGVSSCILTFDKKKTKKTYIDVFKIKNEDICINKFETLEELLKSS  300
WP_003422891.1    201   YKKIIDILKQGGIGSVITPRYFLESLSGKDLREYIKSNVNVQEIVDFLGANIFKNIGVSSCILTFDKKKTKKTYIDVFKIKNEDICINKFEILEELLKSS  300
WP_107616100.1    201   YKKIIDILKQGGIGSVITPRYFLESLSGKDLREYIKSNVNVQEIVDFLGANIFKNIGVSSCILTFDKKKTKKTYIDVFKIKNEDICINKFETLEELLKSS  300
VIF75342.1        201   YKKIIDILKQGGIGSVITPRYFLESLSGKDLREYIKSNVNVQEIVDFLGANIFKNIGVSSCILTFDKKKTKKTYIDVFKIKNEDICINKFETLEELLKSS  300
WP_107619286.1    201   YKKIIDILKQGGIGSVITPRYFLESLSGKDLREYIKSNVNVQEIVDFLGANIFKNIGVSSCILTFDKKKTKKTYIDVFKIKNEDICINKFETLEELLKSS  300
WP_133143045.1    201   YKKIIDILKQGGIGSVITPRYFLESLSGKDLREYIKSNVNVQEIVDFLGANIFKNIGVSSCILTFDKKKTKKTYIDVFKIKNEDICINKFETLEELLKSS  300
EGT2204202.1      201   YKKIIDILKQGGIGSVITPRYFLESLSGKDLREYIKSNVNVQEIVDFLGANIFKNIGVSSCILTFDKKKTKKKYIDVFKIKNEDICINKFETLEELLKSS  300
WP_131040770.1    201   YKKIIDILKQGGIGSVITPRYFLESLSGKDLREYIKSNVNVQEIVDFLGANIFKNIGVSSCILTCDKKKTKKTYIDVFKIKNEDICINKFEILEELLKSS  300
WP_021385685.1    201   YKKIIDILKQGGTGSVITPRYFLESLSGKDLREYIKSNVSVKEIVDFLGANIFKNIGVSSCILTFDKKKSKDKAIDVFKIKNEDICINKFETLEELLNSN  300
WP_054277349.1    201   YKKIIDILKQGGTGSVITPRYFLESLSGKDLREYIKSNVRVKEVVDFLGANIFKNIGVSSCILTFDKKKSKEKSIDVFKIKNEDICINKFETLEELLNSS  300
WP_167640826.1    201   YKKIIDILKQGGTGSVITPRYFLESLSGKDLREYIKSNVRVKEVVDFLGANIFKNIGVSSCILTFDKKKSKEKSIDVFKIKNEDICINKFETLEELLNSS  300
WP_074178811.1    201   YKKIIDILKQGGTGSVITPRYFLESLSGKDLREYIKSNVRVKEVVDFLGANIFKNIGVSSCILTFDKKKSKEKSIDVFKIKNEDICINKFETLEELLNSS  300
NJI79135.1        201   YKKIIDILKQGGTGSVITPRYFLESLSGKDLREYIKSNVRVKEVVDFLGANIFKNIGVSSCILTFDKKKSKDKSIDVFKIKNEDICINKFETLEELLNSS  300
WP_169469916.1    201   YKRIIDILKQGGTGSVITPRYFLESLSGKDLREYIKSNVRVKEIVDFLGANIFKNIGVSSCILTFDKKKSKDKSIDIFKIKNEDICINKFGTLEELLNSS  300
WP_054271332.1    201   YKKIIDILKQGGTGSVITPRYFLESLSGKDLREYIKSNVRVKEVVDFLGANIFKNIGVSSCILTFDKKKSKDKSIDVFKIKNEDICINKFETLEELLNSS  300
EGT4516689.1      201   YKKIIDILKQGGIGSVITPRYFLESLSGKDLREYIKSNVNVQEIVDFLGANIFKNIGVSSCILTFDKKKTKETYIDVFKIKNEDICINKFETLEELLKSS  300
MBH7490777.1      201   YKKIIDILKQGGVGSVITPRYFLESLSGKDLREYIKSNVNVQEIVDFLGANIFKNIGVSSCILTFDKKKTKETYIDVFKIKNEDICINKFETLEELLKSS  300
EGT4155821.1      201   YKKIIDILKQGGIGSVITPRYFLESLSGKDLREYIKSNVNVQEIVDFLGANIFKNIGVSSCILTFDKKKTKETYIDVFKIKNEDICINKFETLEELLKSS  300
MBH7182547.1      201   YKKIIDILKQGGIGSVITPRYFLESLSGKDLREYIKSNVNVQEIVDFLGANIFKNIGVSSCILTFDKKKTKETYIDVFKIKNEDICINKFETLEELLKSS  300
MBF9877285.1      201   YKKIIDILKQGGIGSVITPRYFLESLSGKDLREYIKSNVNVQEIVDFLGANIFKNIGVSSCILTFDKKKTKETYIDVFKIKNEDICINKFETLEELLKSS  300
MBG0179561.1      201   YKKIIDILKQGGIGSVITPRYFLESLSGKDLREYIKSNVNVQEIVDFLGANIFKNIGVSSCILTFDKKKTKETYIDVFKIKNEDICINKFETLEELLKSS  300
MBG0197089.1      201   YKKIIDILKQGGIGSVITPRYFLESLSGKDLREYIKSNVNVQEIVDFLGANIFKNIGVSSCILTFDKKKTKETYIDVFKIKNEDICINKFETLEELLKSS  300
EGT3970705.1      201   YKKIIDILKQGGIGSVITPRYFLESLSGKDLREYIKSNVNVQEIVDFLGANIFKNIGVSSCILTFDKKKTKETYIDVFKIKNEDICINKFETLEELLKSS  300
MBG0131288.1      201   YKKIIDILKQGGIGSVITPRYFLESLSGKDLREYIKSNVNVQEIVDFLGANIFKNIGVSSCILTFDKKKTKETYIDVFKIKNEDICINKFETLEELLKSS  300
EGT4149356.1      201   YKKIIDILKQGGIGSVITPRYFLESLSGKDLREYIKSNVNVQEIVDFLGANIFKNIGVSSCILTFDKKKIKETYIDVFKIKNEDICINKFETLEELLKSS  300
EGT3786504.1      201   YKKIIDILKQGGIGSVITPRYFLESLSGKDLREYIKSNVNVQEIVDFLGANIFKNIGVSSCILTFDKKKTKETYIDVFKIKNEDICINKFETLEELLKSS  300
HAU4954234.1      201   YKKIIDILKQGGIGSVITPRYFLESLSGKDLREYIKSNVNVQEIVDFLGANIFKNIGVSSCILTFDKKKTKETYIDVFKIKNEDICINKFETLEELLKSS  300
WP_131072671.1    201   YKKIIDILKQGGIGSVITPRYFLESLSGKDLREYIKSNVNVQEIVDFLGANIFKNIGVSSCILTFDKKKTKETYIDVFKIKNEDICINKFETLEELLKSS  300
EGT4119714.1      201   YKKIIDILKQGGIGSVITPRYFLESLSGKDLREYIKSNVNVQEIVDFLGANIFKNIGVSSCILTFDKKKTKETYIDVFKIKNEDICINKFETLEELLKSS  300
WP_131036696.1    201   YKKIIDILKQGGIGSVITPRYFLESLSGKDLREYIKSNVNVQEIVDFLGANIFKNIGVSSCILTFDKKKTKETYIDVFKIKNEDICINKFETLEELLKSS  300
EGT5091780.1      201   YKKIIDILKQGGIGSVITPRYFLESLSGKDLREYIKSNVNVQEIVDFLGANIFKNIGVSSCILTFDKKKTKETYIDVFKIKNEDICINKFETLEELLKSS  300
HAT4772283.1      201   YKKIIDILKQGGVGSVITPRYFLESLSGKDLREYIKSNVNVQEIVDFLGANIFKNIGVSSCILTFDKKKTKETYIDVFKIKNEDICINKFETLEELLKSS  300
EGT3843784.1      201   YKKIIDILKQGGIGSVITPRYFLESLSGKDLREYIKSNVNVQEIVDFLGANIFKNIGVSSCILTFDKKKTKETYIDVFKIKNEDICINKFETLEELLKSS  300
WP_095903253.1    201   YKKIIDILKQGGIGSVITPRYFLESLSGKDLREYIKSNVNVQEIVDFLGANIFKNIGVSSCILTFDKKKTKETYIDVFKIKNEDICINKFETLEELLKSS  300
WP_131064843.1    201   YKKIIDILKQGGIGSVITPRYFLESLSGKDLREYIKSNVNVQEIVDFLGANIFKNIGVSSCILTFDKKKTKETHIDVFKIKNEDICINKFETLEELLKSS  300
EGT3983209.1      201   YKKIIDILKQGGIGSVITPRYFLESLSGKDLREYIKSNVNVQEIVDFLGANIFKNIGVSSCILTFDKKKTKETYIDVFKIKNEDICINKFETLEELLKSS  300
HAU5286908.1      201   YKKIIDILKQGGIGSVITPRYFLESLSGKDLREYIKSNVNVQEIVDFLGANIFKNIGVSSCILTFDKKKTKKTYIDVFKIKNEDICINKFETLEELLKSS  300
EGT5172991.1      201   YKKIIDILKQGGIGSVITPRYFLESLSGKDLREYIKSNVNVQEIVDFLGANIFKNIGVSSCILTFDKKKTKETYIDVFKIKNEDICINKFETLEELLKSS  300
WP_021405820.1    201   YKKIIDILKQGGIGSVITPRYFLESLSGKDLREYIKSNVNVQEIVDFLGANIFKNIGVSSCILTFDKKKTKETYIDVFKIKNEDICINKFETLEELLKSS  300
EGT3776579.1      201   YKKIIDILKQGGIGSVITPRYFLESLSGKDLREYIKSNVNVQEIVDFLGANIFKNIGVSSCILTFDKKKTKETYIDVFKIKNEDICINKFETLEELLKSS  300
HAT4798010.1      201   YKKIIDILKQGGVGSVITPRYFLESLSGKDLREYIKSNVNVQEIVDFLGANIFKNIGVSSCILTFDKKKTKETYIDVFKIKNEDICINKFETLEELLKSS  300
HAT4801762.1      201   YKKIIDILKQGGVGSVITPRYFLESLSGKDLREYIKSNVNVQEIVDFLGANIFKNIGVSSCILTFDKKKTKETYIDVFKIKNEDICINKFETLEELLKSS  300
EGT4089306.1      201   YKKIIDILKQGGIGSVITPRYFLESLSGKDLREYIKSNVNVQEIVDFLGANIFKNIGVSSCILTFDKKKTKETYIDVFKIKNEDICINKFETLEELLKSS  300
EGT3949436.1      201   YKKIIDILKQGGIGSVITPRYFLESLSGKDLREYIKSNVNVQEIVDFLGANIFKNIGVSSCILTFDKKKTKETYIDVFKIKNEDICINKFETLEELLKSS  300
EGT4921482.1      201   YKKIIDILKQGGIGSVITPRYFLESLSGKDLREYIKSNVNVQEIVDFLGANIFKNIGVSSCILTFDKKKTKETYIDVFKIKNEDICINKFETLEELLKSS  300
EGT4197963.1      201   YKKIIDILKQGGVGSVITPRYFLESLSGKDLREYIKSNVNVQEIVDFLGANIFKNIGVSSCILTFDKKKTKETYIDVFKIKNEDICINKFETLEELLKSS  300
EGT4973643.1      201   YKKIIDILKQGGIGSVITPRYFLESLSGKDLREYIKSNVNVQEIVDFLGANIFKNIGVSSCILTFDKKKTKETYIDVFKIKNEDICINKFETLEELLKSS  300
WP_095905217.1    201   YKKIIDILKQGGIGSVITPRYFLESLSGKDLREYIKSNVNVQEIVDFLGANIFKNIGVSSCILTFDKKKTKETYIDVFKIKNEDICINKFETLEELLKSS  300
WP_021380625.1    201   YKKIIDILKQGGIGSVITPRYFLESLSGKDLREYIKSNVNVQEIVDFLGANIFKNIGVSSCILTFDKKKTKETYIDVFKIKNEDICINKFETLEELLKSS  300
HAU4836842.1      201   YKKIIDILKQGGIGSVITPRYFLESLSGKDLREYIKSNVNVQEIVDFLGANIFKNIGVSSCILTFDKKKTKETYIDVFKIKNEDICINKFETLEELLKSS  300
WP_167652924.1    201   YKKIIDILKQGGIGSVITPRYFLESLSGKDLREYIKSNVNVQEIVDFLGANIFKNIGVSSCILTFDKKKTKETYIDVFKIKNEDICINKFETLEELLKSS  300
WP_167626365.1    201   YKKIIDILKQGGIGSVITPRYFLESLSGKDLREYIKSNVNVQEIVDFLGANIFKNIGVSSCILTFDKKKTKETYIDVFKIKNEDICINKFETLEELLKSS  300
EGT5068818.1      201   YKKIIDILKQGGIGSVITPRYFLESLSGKDLREYIKSNVNVQEIVDFLGANIFKNIGVSSCILTFDKKKTKETYIDVFKIKNEDICINKFETLEELLKSS  300
WP_131043637.1    201   YKKIIDILKQGGIGSVITPRYFLESLSGKDLREYIKSNVNVQEIVDFLGANIFKNIGVSSCILTFDKKKTKETYIDVFKIKNEDICINKFETLEELLKSS  300
WP_095891409.1    201   YKKIIDILKQGGIGSVITPRYFLESLSGKDLREYIKSNVNVQEIVDFLGANIFKNIGVSSCILTFDKKKTKETYIDVFKIKNEDICINKFETLEELLKSS  300
```

```
                   301       310          320          330          340   TRD>>350         360          370          380          390          400
                    |<<Catalytic Domain|             |            |           b   b|           |            |         ppp         |            |
Query          301  KFEHFNINQRLLSDEWILVNKDDETFYNKIQEKCKYSLEDIAISFQGIITGCDKAFILSKDDVKLNLVDDKFLKCWIKSKNINKYIVDKSEYRLIYSNDI 400
WP_054274745.1 301  KFEHFNINQSLLSDEWILVNKDDETFYNKIQEKCKYSLEDIAISFQGIITGCDKAFILSKDDVKLNLVDDKFLKCWIKSKNINKYIVDKSEYRLIYSNDI 400
VIB38147.1     301  KFEHFNINQRLLSDEWILVNKEDETFYNKIQEKCKYSLEDIAISFQGIITGCDKAFILSKDDVKLNLVDDKFLKCWIKSKNINKYIVDKSEYRLIYSNDI 400
WP_009906092.1 301  KFEHFNINQRLLSDEWILVNKEDETFYNKIQEKCKYSLEDIAISFQGIITGCDKAFILSKDDVKLNLVDDKFLKCWIKSKNINKYVVDKSEYRLIYSNDI 400
WP_003422891.1 301  KFEHFNINQRLLSDEWILVNKEDETFYNKIQEKCKYSLEDIAISFQGIITGCDKAFILSKDDVKLNLVDDKFLKCWIKSKNINKYVVDKSEYRLIYSNDI 400
WP_107616100.1 301  KFEHFNINQRLLSDEWILVNKEDEIFYNKIQEKCKYSLEDIAISFQGIITGCDKAFILSKDDVKLNLVDDKFLKCWIKSKNINKYVVDKSEYRLIYSNDI 400
VIF75342.1     301  KFEHFNINQRLLSDEWILVNKEDEIFYNKIQEKCKYSLEDIAISFQGIITGCDKAFILSKDDVKLNLVDDKFLKCWIKSKNINKYVVDKSEYRLIYSNDI 400
WP_107619286.1 301  KFEHFNINQRLLSDEWILVNKEDEIFYNKIQEKCKYSLEDIAISFQGIITGCDKAFILSKDDVKLNLVDDKFLKCWIKSKNINKYVVDKSDYRLIYSNDI 400
WP_133143045.1 301  KFEHFNINQRLLSDEWILVNKEDEIFYNKIQEKCKYSLEDIAISFQGIITGCDKAFILSKDDVKLNLVDDKFLKCWIKSKNINKYVVDKSEYRLIYSNDI 400
EGT2204202.1   301  KFEHFNINQRLLSDEWILVNKEDEIFYNKIQEKCKYSLEDIAISFQGIITGCDKAFILSKDDVKLNLVDDKFLKCWIKSKNINKYVVDKSEYRLIYSNDI 400
WP_131040770.1 301  KFEHFNINQRLLSDEWILVNKEDETFYNKIQEKCKYSLEDIAISFQGIITGCDKAFILSKDDVKLNLVDDKFLKCWIKSKNINKYVVDKSEYRLIYSNDI 400
WP_021385685.1 301  NFNYFNINQSLLSDEWILVNKDDEAFYNKIQEKCNYSLEDIAISFQGIITGCDKAFILSKDDVKLNLVDNKFLKYWIKSKNINKYIVDKSDYRLIYSNDI 400
WP_054277349.1 301  NFDYFSINQSLLSDEWILVNKADGIFYNKIQEKCNYSLEDIAISFQGIITGCDKAFILSKNDVKLNLVDDKFLKCWIKSKNINKYIVDKSEYRLIYSNDI 400
WP_167640826.1 301  NFDYFSINQSLLSDEWILVNKADGVFYNKIQEKCNYSLEDIAISFQGIITGCDKAFILSKNDVKLNLVDDKFLKCWIKSKNINKYIVDKSEYRLIYSNDI 400
WP_074178811.1 301  NFDYFSINQSLLSDEWILVNKADGVFYNKIQEKCNYSLEDIAISFQGIITGCDKAFILSKNDVKLNLVDDKFLKCWIKSKNINKYIVDKSEYRLIYSNDI 400
NJI79135.1     301  NFDYFSINQSLLSDEWILVNKADGIFYNKIQEKCNYSLEDIAISFQGIITGCDKAFILSKNDVKLNLVDDKFLKCWIKSKNINKYIVDKSEYRLIYSNDI 400
WP_169469916.1 301  NFDYFNINQSLLSDEWILVNKADGVFYNKIQEKCNYSLEDIAISFQGIITGCDKAFILSKNDVKLNLVDDKFLKCWIKSKNINKYTVDKSEYRLIYSNDI 400
WP_054271332.1 301  NFDYFSINQSLLSDEWILVNKADGIFYKKIQEKCNYSLEDIAISFQGIITGCDKAFILSKNDVKLNLVDDKFLKCWIKSKNINKYIVDKSEYRLIYSNDI 400
EGT4516689.1   301  KFEHFNINQRLLSDEWILVNKDDETFYNKIQEKCKYSLEDIAISFQGIITGCDKAFILSKDDVKLNLVDDKFLKCWIKSKNINKYIVDKSEYRLIYSNDI 400
MBH7490777.1   301  KFEHFNINQRLLSDEWILVNKDDETFYNKIQEKCKYSLEDIAISFQGIITGCDKAFILSKDDVKLNLVDDKFLKCWIKSKNINKYIVDKSEYRLIYSNDI 400
EGT4155821.1   301  KFEHFNINQRLLSDEWILVNKDDETFYNKIQEKCKYSLEDIAISFQGIITGCDKAFILSKDDVKLNLVDDKFLKCWIKSKNINKYIVDKSEYRLIYSNDI 400
MBH7182547.1   301  KFEHFNINQRLLSDEWILVNKDDETFYNKIQEKCKYSLEDIAISFQGIITGCDKAFILSKDDVKLNLVDDKFLKCWIKSKNINKYIVDKSEYRLIYSNDI 400
MBF9877285.1   301  KFEHFNINQRLLSDEWILVNKDDETFYNKIQEKCKYSLEDIAISFQGIITGCDKAFILSKDDVKLNLVDDKFLKCWIKSKNINKYIVDKSEYRLIYSNDI 400
MBG0179561.1   301  KFEHFNINQRLLSDEWILVNKDDETFYNKIQEKCKYSLEDIAISFQGIITGCDKAFILSKDDVKLNLVDDKFLKCWIKSKNINKYIVDKSEYRLIYSNDI 400
MBG0197089.1   301  KFEHFNINQRLLSDEWILVNKDDETFYNKIQEKCKYSLEDIAISFQGIITGCDKAFILSKDDVKLNLVDDKFLKCWIKSKNINKYIVDKSEYRLIYSNDI 400
EGT3970705.1   301  KFEHFNINQRLLSDEWILVNKDDETFYNKIQEKCKYSLEDIAISFQGIITGCDKAFILSKDDVKLNLVDDKFLKCWIKSKNINKYIVDKSEYRLIYSNDI 400
MBG0131288.1   301  KFEHFNINQRLLSDEWILVNKDDETFYNKIQEKCKYSLEDIAISFQGIITGCDKAFILSKDDVKLNLVDDKFLKCWIKSKNINKYIVDKSEYRLIYSNDI 400
EGT4149356.1   301  KFEHFNINQRLLSDEWILVNKDDETFYNKIQEKCKYSLEDIAISFQGIITGCDKAFILSKDDVKLNLVDDKFLKCWIKSKNINKYIVDKSEYRLIYSNDI 400
EGT3786504.1   301  KFEHFNINQRLLSDEWILVNKDDETFYNKIQEKCKYSLEDIAISFQGIITGCDKAFILSKDDVKLNLVDDKFLKCWIKSKNINKYIVDKSEYRLIYSNDI 400
HAU4954234.1   301  KFEHFNINQRLLSDEWILVNKDDETFYNKIQEKCKYSLEDIAISFQGIITGCDKAFILSKDDVKLNLVDDKFLKCWIKSKNINKYIVDKSEYRLIYSNDI 400
WP_131072671.1 301  KFEHFNINQRLLSDEWILVNKDDETFYNKIQEKCKYSLEDIAISFQGIITGCDKAFILSKDDVKLNLVDDKFLKCWIKSKNINKYIVDKSEYRLIYSNDI 400
EGT4119714.1   301  KFEHFNITQRLLSDEWILVNKDDETFYNKIQEKCKYSLEDIAISFQGIITGCDKAFILSKDDVKLNLVDDKFLKCWIKSKNINKYIVDKSEYRLIYSNDI 400
WP_131036696.1 301  KFEHFNINQRLLSDEWILVNKDDETFYNKIQEKCKYSLEDIAISFQGIITGCDKAFILSKDDVKLNLVDDKFLKCWIKSKNINKYIVDKSEYRLIYSNDI 400
EGT5091780.1   301  KFEHFNINQRLLSDEWILVNKDDETFYNKIQEKCKYSLEDIAISFQGIITGCDKAFILSKDDVKLNLVDDKFLKCWIKSKNINKYIVDKSEYRLIYSNDI 400
HAT4772283.1   301  KFEHFNINQRLLSDEWILVNKDDETFYNKIQEKCKYSLEDIAISFQGIITGCDKAFILSKDDVKLNLVDDKFLKCWIKSKNINKYIVDKSEYRLIYSNDI 400
EGT3843784.1   301  KFEHFNINQRLLSDEWILVNKDDETFYNKIQEKCKYSLEDIAISFQGIITGCDKAFILSKDDVKLNLVDDKFLKCWIKSKNINKYIVDKSEYRLIYSNDI 400
WP_095903253.1 301  KFEHFNINQRLLSDEWILVNKDDETFYNKIQEKCKYSLEDIAISFQGIITGCDKAFILSKDDVKLNLVDDKFLKCWIKSKNINKYIVDKSEYRLIYSNDI 400
WP_131064843.1 301  KFEHFNINQRLLSDEWILVNKDDETFYNKIQEKCKYSLEDIAISFQGIITGCDKAFILSKDDVKLNLVDDKFLKCWIKSKNINKYIVDKSEYRLIYSNDI 400
EGT3983209.1   301  KFEHFNINQRLLSDEWILVNKDDETFYNKIQEKCKYSLEDIAISFQGIITGCDKAFILSKDDVKLNLVDDKFLKCWIKSKNINKYIVDKSEYRLIYSNDI 400
HAU5286908.1   301  KFEHFNINQRLLSDEWILVNKEDETFYNKIQEKCKYSLEDIAISFQGIITGCDKAFILSKDDVKLNLVDDKFLKCWIKSKNINKYVVDKSEYRLIYSNDI 400
EGT5172991.1   301  KFEHFNINQRLLSDEWILVNKDDETFYNKIQEKCKYSLEDIAISFQGIITGCDKAFILSKDDVKLNLVDDKFLKCWIKSKNINKYIVDKSEYRLIYSNDI 400
WP_021405820.1 301  KFEHFNITQRLLSDEWILVNKDDETFYNKIQEKCKYSLEDIAISFQGIITGCDKAFILSKDDVKLNLVDDKFLKCWIKSKNINKYIVDKSEYRLIYSNDI 400
EGT3776579.1   301  KFEHFNINQRLLSDEWILVNKDDETFYNKIQEKCKYSLEDIAISFQGIITGCDKAFILSKDDVKLNLVDDKFLKCWIKSKNINKYIVDKSEYRLIYSNDI 400
HAT4798010.1   301  KFEHFNINQRLLSDEWILVNKDDETFYNKIQEKCKYSLEDIAISFQGIITGCDKAFILSKDDVKLNLVDDKFLKCWIKSKNINKYIVDKSEYRLIYSNDI 400
HAT4801762.1   301  KFEHFNINQRLLSDEWILVNKDDETFYNKIQEKCKYSLEDIAISFQGIITGCDKAFILSKDDVKLNLVDDKFLKCWIKSKNINKYIVDKSEYRLIYSNDI 400
EGT4089306.1   301  KFEHFNINQRLLSDEWILVNKDDETFYNKIQEKCKYSLEDIAISFQGIITGCDKAFILSKDDVKLNLVDDKFLKCWIKSKNINKYIVDKSEYRLIYSNDI 400
EGT3949436.1   301  KFEHFNINQRLLSDEWILVNKDDETFYNKIQEKCKYSLEDIAISFQGIITGCDKAFILSKDDVKLNLVDDKFLKCWIKSKNINKYIVDKSEYRLIYSNDI 400
EGT4921482.1   301  KFEHFNITQRLLSDEWILVNKDDETFYNKIQEKCKYSLEDIAISFQGIITGCDKAFILSKDDVKLNLVDDKFLKCWIKSKNINKYIVDKSEYRLIYSNDI 400
EGT4197963.1   301  KFEHFNINQRLLSDEWILVNKDDETFYNKIQEKCKYSLEDIAISFQGIITGCDKAFILSKDDVKLNLVDDKFLKCWIKSKNINKYIVDKSEYRLIYSNDI 400
EGT4973643.1   301  KFEHFNINQRLLSDEWILVNKDDETFYNKIQEKCKYSLEDIAISFQGIITGCDKAFILSKDDVKLNLVDDKFLKCWIKSKNINKYIVDKSEYRLIYSNDI 400
WP_095905217.1 301  KFEHFNINQRLLSDEWILVNKDDETFYNKIQEKCKYSLEDIAISFQGIITGCDKAFILSKDDVKLNLVDDKFLKCWIKSKNINKYIVDKSEYRLIYSNDI 400
WP_021380625.1 301  KFEHFNITQRLLSDEWILVNKDDETFYNKIQEKCKYSLEDIAISFQGIITGCDKAFILSKDDVKLNLVDDKFLKCWIKSKNINKYIVDKSEYRLIYSNDI 400
HAU4836842.1   301  KFEHFNINQRLLSDEWILVNKDDETFYNKIQEKCKYSLEDIAISFQGIITGCDKAFILSKDDVKLNLVDDKFLKCWIKSKNINKYIVDKSEYRLIYSNDI 400
WP_167652924.1 301  KFEHFNINQRLLSDEWILVNKDDETFYNKIQEKCKYSLEDIAISFQGIITGCDKAFILSKDDVKLNLVDDKFLKCWIKSKNINKYIVDKSEYRLIYSNDI 400
WP_167626365.1 301  KFEHFNINQRLLSDEWILVNKDDETFYNKIQEKCKYSLEDIAISFQGIITGCDKAFILSKDDVKLNLVDDKFLKCWIKSKNINKYIVDKSEYRLIYSNDI 400
EGT5068818.1   301  KFEHFNINQRLLSDEWILVNKDDETFYNKIQEKCKYSLEDIAISFQGIITGCDKAFILSKDDVKLNLVDDKFLKCWIKSKNINKYIVDKSEYRLIYSNDI 400
WP_131043637.1 301  KFEHFNINQRLLSDEWILVNKDDETFYNKIQEKCKYSLEDIAISFQGIITGCDKAFILSKDDVKLNLVDDKFLKCWIKSKNINKYIVDKSEYRLIYSNDI 400
WP_095891409.1 301  KFEHFNINQRLLSDEWILVNKDDETFYNKIQEKCKYSLEDIAISFQGIITGCDKAFILSKDDVKLNLVDDKFLKCWIKSKNINKYIVDKSEYRLIYSNDI 400
```

```
                      401       410       420       430       440b      450       460       470       480       490       500       510       520
                       |         |       p    b    | p       bb|p        |      bb    |         | bb  p   |         |         |         |p  b  b  |b
Query             401 DNENTNKRILDEIIGLYKTKLENRRECKSGIRKWYELQWGREKLFFERKKIMYPYKSNENRFAIDYDNNFSSADVYSFFIKEEYLDKFSYEYLVGILNSSVYDKYFKITAKKMSKNIYDYYPNKVM 526
WP_054274745.1 401 DNENTNKKVLDEIIGIYKTKLENRRECKSGIRKWYELQWGREKLFFERKKIMYPYKSNENRFAIDYDNNFSSADVYSFFIKEEYLDKFSYEYLVGILNSRIYDKYFKITAKKMSKNIYDYYPNKVM 526
VIB38147.1        401 DNENTNKKILDEIIGIYKTKLENRRECKSGIRKWYELQWGREKLFFERKKIMYPYKSNENRFAIDYDNNFSSADVYSFFIKEEYLGIFSYEYLVGILNSSIYDKYFKITAKKMSKNIYDYYPNKVM 526
WP_009906092.1 401 DNENTNKKILDEIIGIYKTKLENRRECKSGIRKWYELQWGREKLFFERKKIMYPYKSNENRFAIDYDNNFSSADVYSFFIKEEYLGIFSYEYLVGILNSSIYDKYFKITAKKMSKNIYDYYPNKVM 526
WP_003422891.1 401 DNENTNKKILDEIIGIYKTKLENRRECKSGIRKWYELQWGREKLFFERKKIMYPYKSNENRFAIDYDNNFSSADVYSFFIKEEYLGIFSYEYLVGILNSSIYDKYFKITAKKMSKNIYDYYPNKVM 526
WP_107616100.1 401 DNENTNKKILDEIIGIYKTKLENRRECKSGIRKWYELQWGREKLFFERKKIMYPYKSNENRFAIDYDNNFSSADVYSFFIKEEYLGIFSYEYLVGILNSSIYDKYFKITAKKMSKNIYDYYPNKVM 526
VIF75342.1        401 DNENTNKRILDEIIGIYKTKLENRRECKSGIRKWYELQWGREKLFFERKKIMYPYKSNENRFAIDYDNNFSSADVYSFFIKEEYLGIFSYEYLVGILNSSIYDKYFKITAKKMSKNIYDYYPNKVM 526
WP_107619286.1 401 DNENTNKRILDEIIGIYKTKLENRRECKSGIRKWYELQWGREKLFFERKKIMYPYKSNENRFAIDYDNNFSSADVYSFFIKEEYLGIFSYEYLVGILNSSIYDKYFKITAKKMSKNIYDYYPNKVM 526
WP_133143045.1 401 DNENTNKKILDEIIGIYKTKLENRRECKSGIRKWYELQWGREKLFFERKKIMYPYKSNENRFAIDYDNNFSSADVYSFFIKEEYLGIFSYEYLVGILNSSIYDKYFKITAKKMSKNIYDYYPNKVM 526
EGT2204202.1      401 DNENTNKRILDEIIGIYKTKLENRRECKSGIRKWYELQWGREKLFFERKKIMYPYKSNENRFAIDYDNNFSSADVYSFFIKEEYLGIFSYEYLVGILNSSIYDKYFKITAKKMSKNIYDYYPNKVM 526
WP_131040770.1 401 DNENTNKKILDEIIGIYKTKLENRRECKSGIRKWYELQWGREKLFFERKKIMYPYKSNENRFAIDYDNNFSSADVYSFFIKEEYLGIFSYEYLVGILNSSIYDKYFKITAKKMSKNIYDYYPNKVM 526
WP_021385685.1 401 DDEEKNKKILDEIIGIYKSKLENRRECKSGIRKWYELQWGREKLFFERKKIMYPYKSKENRFAIDYDNNFSSADVYSFFIKEEYLDKFSYEYLVGILNSSIYDKYFKITAKKMSKNIYDYYPNKVM 526
WP_054277349.1 401 EDEEKNKKILDEIIGIYKSKLENRRECKNGIRKWYELQWGREKLFFERKKIMYPYKSKENRFAIDYDNNFSSADVYSFFIKEEYLDKFSYEYLVGILNSSIYDKYFKITAKKMSKNIYDYYPNKVM 526
WP_167640826.1 401 EDEEKNKKILDEIIGIYKSKLENRRECKNGIRKWYELQWGREKLFFERKKIMYPYKSKENRFAIDYDNNFSSADVYSFFIKEEYLDKFSYEYLVGILNSSIYDKYFKITAKKMSKNIYDYYPNKVM 526
WP_074178811.1 401 EDEEKNKKILDEIIGIYKSKLENRRECKNGIRKWYELQWGREKLFFERKKIMYPYKSKENRFAIDYDNNFSSADVYSFFIKEEYLDKFSYEYLVGILNSSIYDKYFKITAKKMSKNIYDYYPNKVM 526
NJI79135.1        401 EDEEKNKKILDEIIGIYKSKLENRRECKNGIRKWYELQWGREKLFFERKKIMYPYKSKENRFAIDYDNNFSSADVYSFFIKEEYLDKFSYEYLVGILNSSIYDKYFKITAKKMSKNIYDYYPNKVM 526
WP_169469916.1 401 DNEEENKKILDEIIGIYKSKLENRRECKNGIRKWYELQWGREKLFFERKKIMYPYKSKENRFAIDYDNNFSSADVYSFFIKEEYLDKFSYEYLVGILNSSIYDKYFKITAKKMSKNIYDYYPNKVM 526
WP_054271332.1 401 EDEEKNKKILDEIIGIYKSKLENRRECKNGIRKWYELQWGREKLFFERKKIMYPYKSKENRFAIDYDNNFSSADVYSFFIKEEYLDKFSYEYLVGILNSSIYDKYFKITAKKMSKNIYDYYPNKVM 526
EGT4516689.1      401 DNENTNKRILDEIIGLYKTKLENRRECKSGIRKWYELQWGREKLFFERKKIMYPYKSNENRFAIDYDNNFSSADVYSFFIKEEYLDKFSYEYLVGILNSSVYDKYFKITAKKMSKNIYDYYPNKVM 526
MBH7490777.1      401 DNENTNKKILDEIIGLYKTKLENRRECKSGIRKWYELQWGREKLFFERKKIMYPYKSNENRFAIDYDNNFSSADVYSFFIKEEYLDKFSYEYLVGILNSSVYDKYFKITAKKMSKNIYDYYPNKVM 526
EGT4155821.1      401 DNENTNKRILDEIIGLYKTKLENRRECKSGIRKWYELQWGREKLFFERKKIMYPYKSNENRFAIDYDNNFSSADVYSFFIKEEYLDKFSYEYLVGILNSSVYDKYFKITAKKMSKNIYDYYPNKVM 526
MBH7182547.1      401 DNENTNKRILDEIIGLYKTKLENRRECKSGIRKWYELQWGREKLFFERKKIMYPYKSNENRFAIDYDNNFSSADVYSFFIKEEYLDKFSYEYLVGILNSSVYDKYFKITAKKMSKNIYDYYPNKVM 526
MBF9877285.1      401 DNENTNKRILDEIIGLYKTKLENRRECKSGIRKWYELQWGREKLFFERKKIMYPYKSNENRFAIDYDNNFSSADVYSFFIKEEYLDKFSYEYLVGILNSSVYDKYFKITAKKMSKNIYDYYPNKVM 526
MBG0179561.1      401 DNENTNKRILDEIIGLYKTKLENRRECKSGIRKWYELQWGREKLFFERKKIMYPYKSNENRFAIDYDNNFSSADVYSFFIKEEYLDKFSYEYLVGILNSSVYDKYFKITAKKMSKNIYDYYPNKVM 526
MBG0197089.1      401 DNENTNKRILDEIIGLYKTKLENRRECKSGIRKWYELQWGREKLFFERKKIMYPYKSNENRFAIDYDNNFSSADVYSFFIKEEYLDKFSYEYLVGILNSSVYDKYFKITAKKMSKNIYDYYPNKVM 526
EGT3970705.1      401 DNENTNKRILDEIIGLYKTKLENRRECKSGIRKWYELQWGREKLFFERKKIMYPYKSNENRFAIDYDNNFSSADVYSFFIKEEYLDKFSYEYLVGILNSSVYDKYFKITAKKMSKNIYDYYPNKVM 526
MBG0131288.1      401 DNENTNKRILDEIIGLYKTKLENRRECKSGIRKWYELQWGREKLFFERKKIMYPYKSNENRFAIDYDNNFSSADVYSFFIKEEYLDKFSYEYLVGILNSSVYDKYFKITAKKMSKNIYDYYPNKVM 526
EGT4149356.1      401 DNENTNKRILDEIIGLYKTKLENRRECKSGIRKWYELQWGREKLFFERKKIMYPYKSNENRFAIDYDNNFSSADVYSFFIKEEYLDKFSYEYLVGILNSSVYDKYFKITAKKMSKNIYDYYPNKVM 526
EGT3786504.1      401 DNENTNKRILDEIIGLYKTKLENRRECKSGIRKWYELQWGREKLFFERKKIMYPYKSNENRFAIDYDNNFSSADVYSFFIKEEYLDKFSYEYLVGILNSSVYDKYFKITAKKMSKNIYDYYPNKVM 526
HAU4954234.1      401 DNENTNKRILDEIIGVYKTKLENRRECKSGIRKWYELQWGREKLFFERKKIMYPYKSNENRFAIDYDNNFSSADVYSFFIKEEYLDKFSYEYLVGILNSSVYDKYFKITAKKMSKNIYDYYPNKVM 526
WP_131072671.1 401 DNENTNKRILDEIIGVYKTKLENRRECKSGIRKWYELQWGREKLFFERKKIMYPYKSNENRFAIDYDNNFSSADVYSFFIKEEYLDKFSYEYLVGILNSSVYDKYFKITAKKMSKNIYDYYPNKVM 526
EGT4119714.1      401 DNENTNKRILDEIIGLYKTKLENRRECKSGIRKWYELQWGREKLFFERKKIMYPYKSNENRFAIDYDNNFSSADVYSFFIKEEYLDKFSYEYLVGILNSSVYDKYFKITAKKMSKNIYDYYPNKVM 526
WP_131036696.1 401 DNENTNKRILDEIIGVYKTKLENRRECKSGIRKWYELQWGREKLFFERKKIMYPYKSNENRFAIDYDNNFSSADVYSFFIKEEYLDKFSYEYLVGILNSSVYDKYFKITAKKMSKNIYDYYPNKVM 526
EGT5091780.1      401 DNENTNKRILDEIIGLYKTKLENRRECKSGIRKWYELQWGREKLFFERKKIMYPYKSNENRFAIDYDNNFSSADVYSFFIKEEYLDKFSYEYLVGILNSSVYDKYFKITAKKMSKNIYDYYPNKVM 526
HAT4772283.1      401 DNENTNKKILDEIIGLYKTKLENRRECKSGIRKWYELQWGREKLFFERKKIMYPYKSNENRFAIDYDNNFSSADVYSFFIKEEYLDKFSYEYLVGILNSSVYDKYFKITAKKMSKNIYDYYPNKVM 526
EGT3843784.1      401 DNENTNKRILDEIIGLYKTKLENRRECKSGIRKWYELQWGREKLFFERKKIMYPYKSNENRFAIDYDNNFSSADVYSFFIKEEYLDKFSYEYLVGILNSSVYDKYFKITAKKMSKNIYDYYPNKV 525
WP_095903253.1 401 DNENTNKRILDEIIGLYKTKLENRRECKSGIRKWYELQWGREKLFFERKKIMYPYKSNENRFAIDYDNNFSSADVYSFFIKEEYLDKFSYEYLVGILNSSVYDKYFKITAKKMSKNIYDYYPNK 524
WP_131064843.1 401 DNENTNKRILDEIIGLYKTKLENRRECKSGIRKWYELQWGREKLFFERKKIMYPYKSNENRFAIDYDNNFSSADVYSFFIKEEYLDKFSYEYLVGILNSSVYDKYFKITAKKMSKNIYDYYP 522
EGT3983209.1      401 DNENTNKRILDEIIGLYKTKLENRRECKSGIRKWYELQWGREKLFFERKKIMYPYKSNENRFAIDYDNNFSSADVYSFFIKEEYLDKFSYEYLVGILNSSVYDKYFKITAKKMSKNIYD 519
HAU5286908.1      401 DNENTNKKILDEIIGIYKTKLENRRECKSGIRKWYELQWGREKLFFERKKIMYPYKSNENRFAIDYDNNFSSADVYSFFIKEEYLGIFSYEYLVGILNSSIYDKYFKITAKKMSKNIYD 519
EGT5172991.1      401 DNENTNKRILDEIIGLYKTKLENRRECKSGIRKWYELQWGREKLFFERKKIMYPYKSNENRFAIDYDNNFSSADVYSFFIKEEYLDKFSYEYLVGILNSSVYDKYFKITAKKMS 514
WP_021405820.1 401 DNENTNKRILDEIIGLYKTKLENRRECKSGIRKWYELQWGREKLFFERKKIMYPYKSNENRFAIDYDNNFSSADVYSFFIKEEYLDKFSYEYLVGILNSSVYDKYFKITAKK 512
EGT3776579.1      401 DNENTNKRILDEIIGLYKTKLENRRECKSGIRKWYELQWGREKLFFERKKIMYPYKSNENRFAIDYDNNFSSADVYSFFIKEEYLDKFSYEYLVGILNSSVYDKYFKITAK 511
HAT4798010.1      401 DNENTNKKILDEIIGLYKTKLENRRECKSGIRKWYELQWGREKLFFERKKIMYPYKSNENRFAIDYDNNFSSADVYSFFIKEEYLDKFSYEYLVGILNSSVYDKYFKITAK 511
HAT4801762.1      401 DNENTNKKILDEIIGLYKTKLENRRECKSGIRKWYELQWGREKLFFERKKIMYPYKSNENRFAIDYDNNFSSADVYSFFIKEEYLDKFSYEYLVGILNSSVYDKYFKITA 510
EGT4089306.1      401 DNENTNKRILDEIIGLYKTKLENRRECKSGIRKWYELQWGREKLFFERKKIMYPYKSNENRFAIDYDNNFSSADVYSFFIKEEYLDKFSYEYLVGILNSSVYDKYFKIT 509
EGT3949436.1      401 DNENTNKRILDEIIGVYKTKLENRRECKSGIRKWYELQWGREKLFFERKKIMYPYKSNENRFAIDYDNNFSSADVYSFFIKEEYLDKFSYEYLVGILNSSVYDKYFKIT 509
EGT4921482.1      401 DNENTNKRILDEIIGLYKTKLENRRECKSGIRKWYELQWGREKLFFERKKIMYPYKSNENRFAIDYDNNFSSADVYSFFIKEEYLDKFSYEYLVGILNSSVYDKYFKI 508
EGT4197963.1      401 DNENTNKKILDEIIGLYKTKLENRRECKSGIRKWYELQWGREKLFFERKKIMYPYKSNENRFAIDYDNNFSSADVYSFFIKEEYLDKFSYEYLVGILNSSVYDKYFKI 508
EGT4973643.1      401 DNENTNKRILDEIIGLYKTKLENRRECKSGIRKWYELQWGREKLFFERKKIMYPYKSNENRFAIDYDNNFSSADVYSFFIKEEYLDKFSYEYLVGILNSSVYDKYFK 507
WP_095905217.1 401 DNENTNKRILDEIIGLYKTKLENRRECKSGIRKWYELQWGREKLFFERKKIMYPYKSNENRFAIDYDNNFSSADVYSFFIKEEYLDKFSYEYLVGILNSSVYDKY 505
WP_021380625.1 401 DNENTNKRILDEIIGLYKTKLENRRECKSGIRKWYELQWGREKLFFERKKIMYPYKSNENRFAIDYDNNFSSADVYSFFIKEEYLDKFSYEYLVGILNSSVY 502
HAU4836842.1      401 DNENTNKRILDEIIGLYKTKLENRRECKSGIRKWYELQWGREKLFFERKKIMYPYKSNENRFAIDYDNNFSSADVYSFFIKEEYLDKFSYEYLVGILNSSV 501
WP_167652924.1 401 DNENTNKRILDEIIGLYKTKLENRRECKSGIRKWYELQWGREKLFFERKKIMYPYKSNENRFAIDYDNNFSSADVYSFFIKEEYLDKFSYEYLVGILNSS 500
WP_167626365.1 401 DNENTNKRILDEIIGLYKTKLENRRECKSGIRKWYELQWGREKLFFERKKIMYPYKSNENRFAIDYDNNFSSADVYSFFIKEEYLDKFSYEYLVGIL 497
EGT5068818.1      401 DNENTNKRILDEIIGLYKTKLENRRECKSGIRKWYELQWGREKLFFERKKIMYPYKSNENRFAIDYDNNFSSADVYSFFIKEEY 484
WP_131043637.1 401 DNENTNKRILDEIIGVYKTKLENRRECKSGIRKWYELQWGREKLFFERKKIMYPYKSNENRFAIDYDNNFSSADVYSFFI 480
WP_095891409.1 401 DNENTNKRILDEIIGLYKTKLENRRECKSGIRKWYELQWGREKLFFERKKIMYPYKSNENRFAIDY 466
```

```
                     530       540       550       560       570
                       |         |         |         |         |
Query           527 KIRIFRDNNYEEIENLSKQIISILLNKSIDKGKVEKLQIKMDNLIMDSLGI 577
WP_054274745.1 527 KIRIFRDNNYEEIENLSKQIISILLDKSIDKEKVERLQIKMDNLIMDSLGI 577
VIB38147.1     527 KIRIFRDNNYDEIENLSKQIISILLNKSIDKGKVEKLQIKMDNLIMDSLGI 577
WP_009906092.1 527 KIRIFRDNNYDEIENLSKQIISILLNKSIDKGKVEKLQIKMDNLIMDSLGI 577
WP_003422891.1 527 KIRIFRDNNYDEIENLSKQIISILLNKSIDKGKVEKLQIKMDNLIMDSLGI 577
WP_107616100.1 527 KIRIFRDNNYDEIENLSKQIISILLNKSIDKGKVEKLQIKMDNLIMDSLGI 577
VIF75342.1     527 KIRIFRDNNYDEIENLSKQIISILLNKSIDKGKVEKLQIKMDNLIMDSLGI 577
WP_107619286.1 527 KIRIFRDNNYDEIENLSKQIISILLNKSIDKGKVEKLQIKMDNLIMDSLGI 577
WP_133143045.1 527 KIRIFRDNNYDEIENLSKQIISILLNKSIDKGKVEKLQIKMDNLIMDSLGI 577
EGT2204202.1   527 KIRIFRDNNYDEIENLSKQIISILLNKSTDKEKVEKLQIKMDNLIMDSLGI 577
WP_131040770.1 527 KIRIFRDNNYDEIENLSKQIISILLNKSIDKGKVEKLQIKMDNLIMDSLGI 577
WP_021385685.1 527 KIRIFRDNNYEEIESLSRQIISILLNKSIDKEKVERLQIKMDNLIIDSLGI 577
WP_054277349.1 527 KIRIFRDNNYEEIESLSKEIISILLNNSVDKEKVERLQIKMDNLIMNSLGI 577
WP_167640826.1 527 KIRIFRDNNYEEIESLSKEIISILLNNSVDKEKIERLQIKMDNLIMNSLGI 577
WP_074178811.1 527 KIRIFRDNNYEEIESLSKEIISILLNNSVDKEKIERLQIKMDNLIMNSLGI 577
NJI79135.1     527 KIRIFRDNNYEEIESLSKEIISILLNNSVDKEKIERLQIKMDNLIMNSLGI 577
WP_169469916.1 527 KIRIFRDNNYEEIESLSKQIISILLNNSIDKEKVEMLQIKMDNLIIDSLGI 577
WP_054271332.1 527 KIRIFRDNNYEEIESLSKEIISILLNNSVDKEKIERLQIKMDNLIMNSLGI 577
EGT4516689.1   527 KIRIFRDNNYEEIENLSKQIISILLNKSIDKGKVEKLQIKMDNLI 571
MBH7490777.1   527 KIRIFRDNNYEEIENLSKQIISILLNKSIDKGKVEKLQIKMDNLI 571
EGT4155821.1   527 KIRIFRDNNYEEIENLSKQIISILLNKSIDKGKVEKLQIKMDN 569
MBH7182547.1   527 KIRIFRDNNYEEIENLSKQIISILLNKSIDKGKVEKLQ 564
MBF9877285.1   527 KIRIFRDNNYEEIENLSKQIISILLNKSIDKGKVEKL 563
MBG0179561.1   527 KIRIFRDNNYEEIENLSKQIISILLNKSIDKGKVEK 562
MBG0197089.1   527 KIRIFRDNNYEEIENLSKQIISILLNKSIDKGKVE 561
EGT3970705.1   527 KIRIFRDNNYEEIENLSKQIISILLNKSIDKGKV 560
MBG0131288.1   527 KIRIFRDNNYEEIENLSKQIISILLNKSIDKG 558
EGT4149356.1   527 KIRIFRDNNYEEIENLSKQIISILLNKSIDK 557
EGT3786504.1   527 KIRIFRDNNYEEIENLSKQI 546
HAU4954234.1   527 KIRIFRDNNYEEIE 540
WP_131072671.1 527 KIRIFRD 533
EGT4119714.1   527 KIRIFR 532
WP_131036696.1 527 KIRI 530
EGT5091780.1   527 KI 528
```
