# Supplementary material for: Clostridioides difficile specific DNA adenine methyltransferase CamA squeezes and flips adenine out of DNA helix
Source: Nat Commun. 2021 Jun 8;12:3436. doi: 10.1038/s41467-021-23693-w (PMC8187626; doi:10.1038/s41467-021-23693-w)
Supplement: Supplementary file 3 — Reporting summary [file 41467_2021_23693_MOESM3_ESM.pdf]

nature research

Corresponding author(s): Xing Zhang and Xiaodong Cheng

Last updated by author(s): May 7, 2021

Reporting Summary

Nature Research wishes to improve the reproducibility of the work that we publish. This form provides structure for consistency and transparency in reporting. For further information on Nature Research policies, see our Editorial Policies and the Editorial Policy Checklist.

Statistics

For all statistical analyses, confirm that the following items are present in the figure legend, table legend, main text, or Methods section.

n/a	Confirmed	
☐	☒	The exact sample size (n) for each experimental group/condition, given as a discrete number and unit of measurement
☐	☒	A statement on whether measurements were taken from distinct samples or whether the same sample was measured repeatedly
☒	☐	The statistical test(s) used AND whether they are one- or two-sided<br>*Only common tests should be described solely by name; describe more complex techniques in the Methods section.*
☒	☐	A description of all covariates tested
☒	☐	A description of any assumptions or corrections, such as tests of normality and adjustment for multiple comparisons
☐	☒	A full description of the statistical parameters including central tendency (e.g. means) or other basic estimates (e.g. regression coefficient) AND variation (e.g. standard deviation) or associated estimates of uncertainty (e.g. confidence intervals)
☒	☐	For null hypothesis testing, the test statistic (e.g. F, t, r) with confidence intervals, effect sizes, degrees of freedom and P value noted<br>*Give P values as exact values whenever suitable.*
☒	☐	For Bayesian analysis, information on the choice of priors and Markov chain Monte Carlo settings
☒	☐	For hierarchical and complex designs, identification of the appropriate level for tests and full reporting of outcomes
☒	☐	Estimates of effect sizes (e.g. Cohen's d, Pearson's r), indicating how they were calculated

Our web collection on statistics for biologists contains articles on many of the points above.

Software and code

Policy information about availability of computer code

Data collection	X-ray diffraction data were collected from synchrotron beamline SERCAT (22-ID) at Advanced Photon Source of Argonne National Laboratory
Data analysis	Crystallographic datasets were first processed with HKL2000 (version v719.2).<br>PHENIX (version 1.19_4092) Xtriage was used to analyze Se-met anomalous signals; PHENIX AutoSol module generated an initial model.<br>PHENIX AutoBuild were used for initial model building; PHENIX Refine was used for refinement.<br>COOT (0.8.9.3-pre EL revision 8011) was used for manual building of structure model and corrections between refinement rounds.<br>PyMol (version 2.3.5) was used for graphics.<br>Structure quality was analyzed during rounds of PHENIX refinements and validated by the PDB validation server

For manuscripts utilizing custom algorithms or software that are central to the research but not yet described in published literature, software must be made available to editors and reviewers. We strongly encourage code deposition in a community repository (e.g. GitHub). See the Nature Research guidelines for submitting code & software for further information.

April 2020

Data

Policy information about availability of data

All manuscripts must include a data availability statement. This statement should provide the following information, where applicable:

- Accession codes, unique identifiers, or web links for publicly available datasets
- A list of figures that have associated raw data
- A description of any restrictions on data availability

The X-ray structure (coordinates) and the source data (structure factor file) of CamA with bound DNA have been submitted to the PDB under accession numbers 7LNI (SeMet-CamA+DNA), 7LNJ (CamA+DNA) and 7LT5 (CamA+DNA+SAH).
Source data are provided for Figure 1, panels A and B, Figure 3, panels H and I, and Supplementary Figure S1, panels B to H.

Field-specific reporting

Please select the one below that is the best fit for your research. If you are not sure, read the appropriate sections before making your selection.

[x] Life sciences [ ] Behavioural & social sciences [ ] Ecological, evolutionary & environmental sciences

For a reference copy of the document with all sections, see nature.com/documents/nr-reporting-summary-flat.pdf

Life sciences study design

All studies must disclose on these points even when the disclosure is negative.

Sample size	Variations were provided for each experiment as long as the enzyme activity stays within the linear range:<br>SAH concentrations 1-4 uM (Figure S1B), pH 5.5-8.8 (Figure S1C), NaCl concentrations 0-175 mM (Figure S1D), reaction time 20-180 sec (Figure S1E), enzyme concentrations 12.5-200 nM (Figure S1F), incubation time 2.5-20 min (Figure S1G), temperature (Figure S1H), SAM concentrations 1.25-40 uM (Figure 1A), DNA concentrations 6.26 nM-1 uM (Figure 1B).
Data exclusions	None.
Replication	Co-Crystallizations were repeated (N=3) for wild-type CamA and SeMet-substituted CamA, as well as for CamA-DNA complex in the absence and presence of SAH.
Randomization	Structure refinements were performed with 5% randomly chosen reflections for validation by R-free values.<br>No randomization was necessary as only single variable changed per enzymatic experiment.
Blinding	Blinding was not needed, as there were no experiments requiring subjective interpretation. X-ray diffraction data and enzymatic data were measured quantitatively. Positive and negative controls were included in each measurement for single variable parameter.

Reporting for specific materials, systems and methods

We require information from authors about some types of materials, experimental systems and methods used in many studies. Here, indicate whether each material, system or method listed is relevant to your study. If you are not sure if a list item applies to your research, read the appropriate section before selecting a response.

Materials & experimental systems

n/a	Involved in the study	
[x]	[ ]	Antibodies
[x]	[ ]	Eukaryotic cell lines
[x]	[ ]	Palaeontology and archaeology
[x]	[ ]	Animals and other organisms
[x]	[ ]	Human research participants
[x]	[ ]	Clinical data
[x]	[ ]	Dual use research of concern

Methods

n/a	Involved in the study	
[x]	[ ]	ChIP-seq
[x]	[ ]	Flow cytometry
[x]	[ ]	MRI-based neuroimaging
